# Full-Fiber Auxetic-Interlaced Yarn Sensor for Sign-Language Translation Glove Assisted by Artificial Neural Network

**DOI:** 10.1007/s40820-022-00887-5

**Published:** 2022-07-01

**Authors:** Ronghui Wu, Sangjin Seo, Liyun Ma, Juyeol Bae, Taesung Kim

**Affiliations:** 1 Department of Mechanical Engineering, Ulsan National Institute of Science and Technology (UNIST), 50 UNIST-Gil, Ulsan, 44919 Republic of Korea; 2 College of Physical Science and Technology, Xiamen University, Xiamen, 361005 People’s Republic of China

**Keywords:** Negative Poisson’s ratio yarns, Interlaced yarn sensors, Smart glove, Deep learning, Sign-language translation

## Abstract

Yarn sensors have shown promising application prospects in wearable electronics owing to their shape adaptability, good flexibility, and weavability. However, it is still a critical challenge to develop simultaneously structure stable, fast response, body conformal, mechanical robust yarn sensor using full microfibers in an industrial-scalable manner. Herein, a full-fiber auxetic-interlaced yarn sensor (AIYS) with negative Poisson’s ratio is designed and fabricated using a continuous, mass-producible, structure-programmable, and low-cost spinning technology. Based on the unique microfiber interlaced architecture, AIYS simultaneously achieves a Poisson’s ratio of−1.5, a robust mechanical property (0.6 cN/dtex), and a fast train-resistance responsiveness (0.025 s), which enhances conformality with the human body and quickly transduce human joint bending and/or stretching into electrical signals. Moreover, AIYS shows good flexibility, washability, weavability, and high repeatability. Furtherly, with the AIYS array, an ultrafast full-letter sign-language translation glove is developed using artificial neural network. The sign-language translation glove achieves an accuracy of 99.8% for all letters of the English alphabet within a short time of 0.25 s. Furthermore, owing to excellent full letter-recognition ability, real-time translation of daily dialogues and complex sentences is also demonstrated. The smart glove exhibits a remarkable potential in eliminating the communication barriers between signers and non-signers.

**Supplementary Information:**

The online version contains supplementary material available at 10.1007/s40820-022-00887-5.

## Highlights

- Full-fiber auxetic-interlaced yarn sensor was fabricated by a continuous and mass-producible computerized wrapping spinning technology.
- Auxetic-interlaced yarn sensor shows a Poisson’s ratio of − 1.5, a robust mechanical property (0.6 cN/dtex), and a fast train-resistance responsiveness (0.025 s).
- A novel sign-language translation glove was developed to recognize the full English alphabet and translate the wearer’s sign language to text.

**Supplementary Information:**

The online version contains supplementary material available at 10.1007/s40820-022-00887-5.

## Graphical abstract

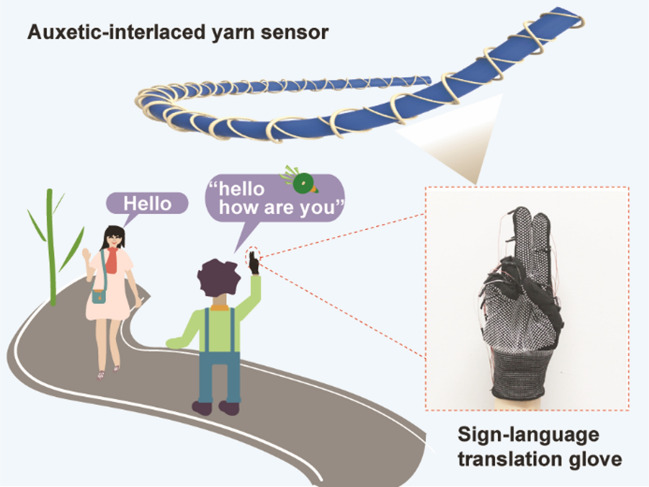

## Introduction

Hearing-impaired people can only rely on sign language to communicate and exchange ideas with the world [1, 2]. Thus, portable and flexible sign-language translation systems that can translate gestures into text or voice are clearly needed. For the English language, such a system is required to recognize all 26 letters of the English alphabet with high accuracy in any environment. Currently available sign-language translation systems can be divided into two categories: vision-based system [3] and sensor-based gloves [4, 5] such as electromyography [6–8], pressure sensor [9], and strain/stress sensors [5, 10, 11]. The vision-based approach requires strict imaging condition requirements, such as camera angle [12], illumination [13], and background [1, 3, 13]. This makes the vision-based approach impractical in the complex and variable daily life of deaf people. In these scenarios, sensor-based wearables show more potential because of their strong anti-interference ability to the environment. However, sensor-based systems are still limited by many issues, such as small amount of recognizable sign numbers [7, 14], long translation time [10, 14], structural complexity [10], and lack of conformality [14]. Moreover, strict adherence to requirements for the form-factor, sensitivity, resolution, and mechanical compliance is highly needed for glove sensors to achieve sign-language translation.

Yarn sensors [2, 15–20] provide a new alternative approach for wearable sign-language translation because such textiles can be compatible with traditional textile-production processes [21] and have the functionality of detecting human joint motion [22–27]. Unlike film-based sensors [9, 10, 28–30] that are difficult to integrate into wearable textile gloves, full-fiber yarn sensors are flexible, invisible, and breathable in wearable clothes or gloves [31–33]. However, the further advancement of yarn sensors still faces several critical challenges. First, current yarn sensors normally have core-shell structures with unidirectional twists [34–38]. This architecture only allows fibers assembled in one twisting direction, which results in structure instability because of the directional residual torque in the spinning process. Second, the fabrication of yarn sensors, such as emulsion dipping, composite laminating, and sputter coating, is difficult to maintain an even surface because of the Plateau-Rayleigh instability [39]. The non-fiber uneven yarns will restrict the flexibility, breathability, and stretchability of the fabric sensors [40]. Third, many yarn sensors are not compatible with traditional textile-production processes, therefore have difficulties in mass-production and structure manipulation [41]. Forth, the current yarn sensors normally have a positive Poisson’s ratio [34–38], which will contract in the yarn axial directions when it is stretched longitudinally. This may result in stress concentration and restricts the future study in conformality with human bending joint parts [42, 43].

To address the aforementioned problems, we report a full-fiber auxetic-interlaced yarn sensor (AIYS) using a continuous, mass-producible spinning technology. Two conductive polyamide (PA) yarns are interlocked with the core polyurethane (PU) yarn along the wrapping direction at a high speed. Furthermore, the geometric and auxetic behavior, mechanical properties, and electrical performance of the AIYS during stretching are analyzed. Moreover, we propose a new mechanical constitutive model that fully considers the structure distribution and nonlinear mechanical behavior of the AIYS, which shows a high consistence with the experimental data. In addition, a smart glove sewed with a 16-AIYS array covering the entire movable joint of the human hand and wrist is fabricated. An artificial neural network (ANN) algorithm was developed for sensor calibration and correction. We demonstrate that the sign-language translation glove has an overall recognition accuracy of 99.8% for the 26 letters of the English alphabet, according to American Sign Language (ASL) [18]. Moreover, the smart glove makes it possible to transduce human thoughts from sign language into text or voice with the aid of mobile devices at a rapid speed. Therefore, our low-cost, full-fiber, mass-producible sign-language translation glove with excellent flexibility, high recognition accuracy, and good body conformality will be helpful for the hearing-impaired community.

## Experimental Section

### Materials

Conductive silver-coated PA yarns were purchased from Qingdao Zhiyuan Xiangyu Functional Fabric Co., Ltd., China. PU yarns were purchased from Huaian New Technology Co., Ltd., China. Knitted gloves were purchased from Hwa Heung Glove Company, South Korea.

### Preparation of the AIYS and Smart Glove

**Fig. 1:**
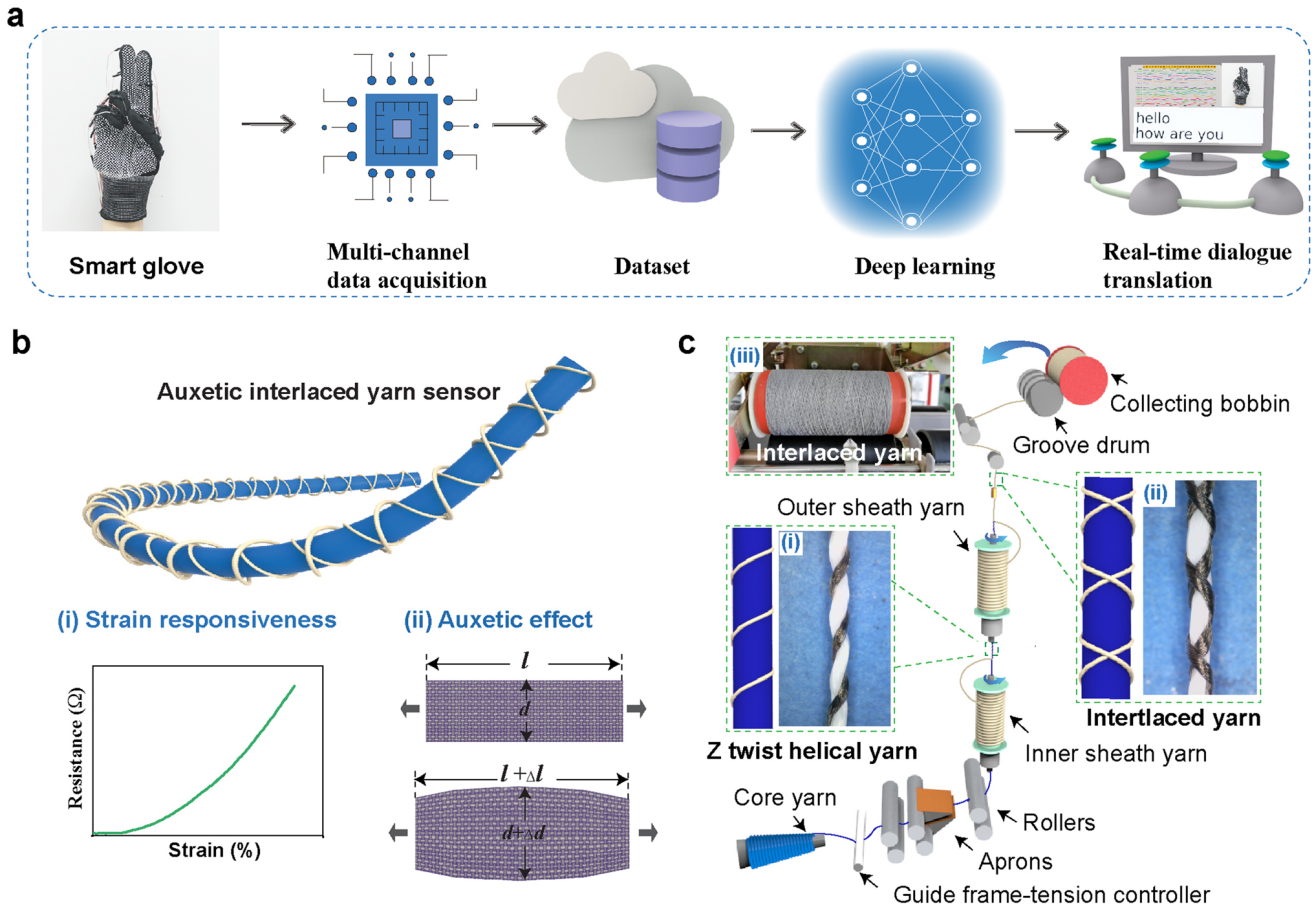
Schematic illustration of the sign-language translation glove based on auxetic yarn sensor array. **a** Diagram of the real-time sign-language translation, showing signal acquisition, data processing, and deep learning paths from the AIYS-array-embedded smart glove to the sign-language translation. **b** Architecture of the AIYS and illustration of its strain responsiveness and auxetic effect. **c** Schematic illustration of the continuous yarn-wrapping technology. Insets (i) and (ii) are optical images of the Z-twist helical yarn and interlaced-helical yarn, and (iii) is a picture of collecting bobbins with the AIYS

**Fig. 2:**
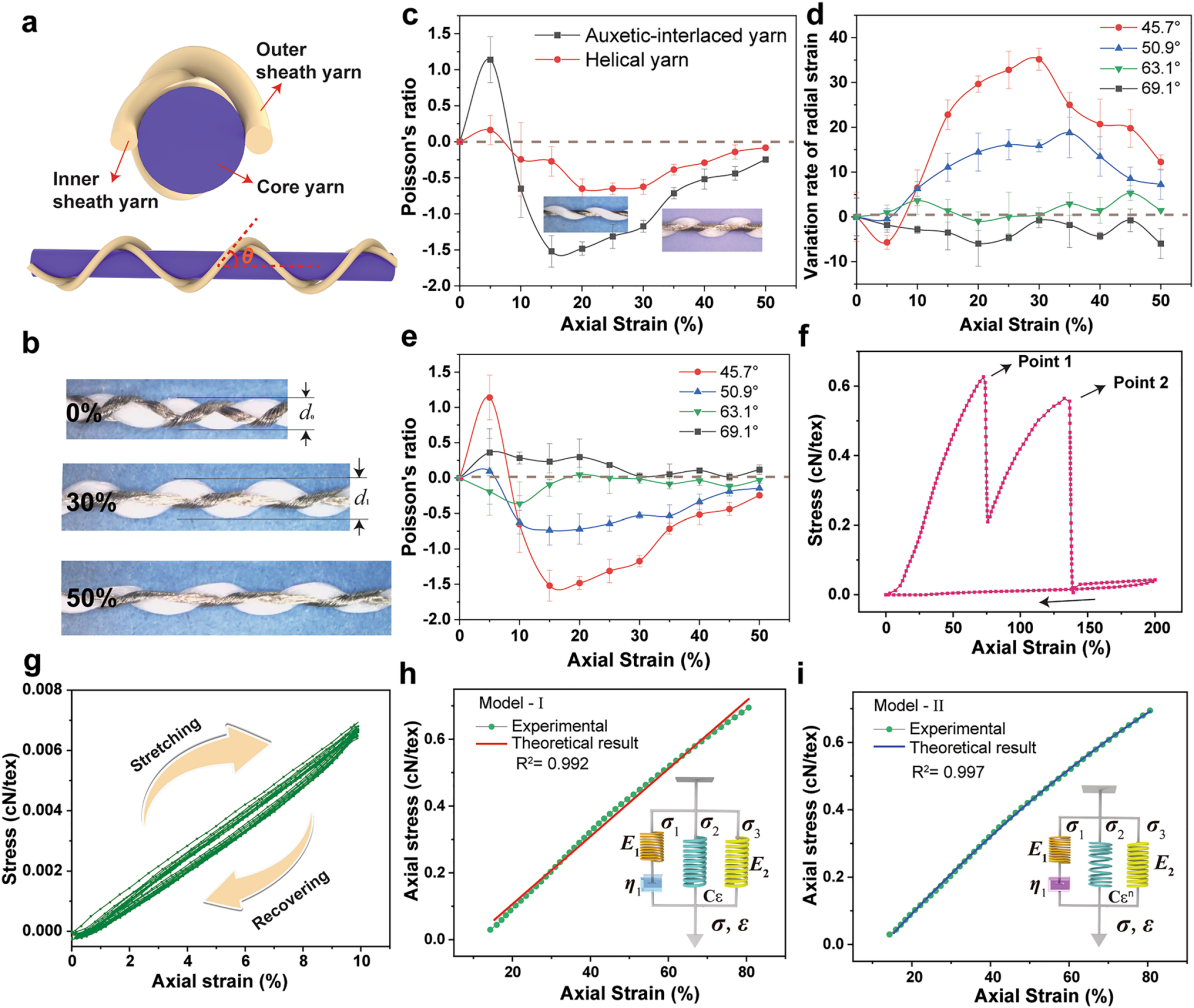
Geometric and mechanical behavior of an AIYS sensing unit. **a** Cross-sectional and side view of the AIYS. **b** AIYS being stretched to 0%, 30%, and 50% elongation, showing negative Poisson’s ratio behavior. **c** Comparison of the Poisson’s ratios between interlaced-helical and single-helical auxetic yarn. “Axial strain” means the stretching strain applied along the axial direction of AIYS composite yarn. **d** Variation of the radial strains of AIYS with different wrapping angles of 45.7°, 50.9°, 63.1°, 69.1°. “Radial strain” means the deformation strain measured along the radial direction of AIYS composite yarn. **e** Poisson’s ratios of the AIYS with different wrapping angles. **f** Typical mechanical behavior of the AIYS during stretching and recovery from 0 to 200% elongation. **g** Cyclic mechanical stretching performance of the AIYS during 0–10% elongation. **h** Comparison of experimental and theoretical viscoelastic mechanical behavior of an AIYS described by Model-I and Model-II

AIYS was fabricated using the JGC141 fully computerized yarn-wrapping machine, which was purchased from Zhejiang Jingong Science and Technology Co., Ltd, Zhejiang, China. First, the silver-coated PA yarn for the inner sheath layer was transferred from a commercial bobbin to a hollow yarn bobbin through a yarn-pressing machine in a clockwise direction. Second, the process was conducted in a counterclockwise direction for the outer sheath layer bobbin. Third, the two hollow yarn bobbins with the silver-coated PA yarns were mounted on the fully computerized yarn-wrapping machine in a proper order. Fourth, as shown in Fig. 2, the core yarn was placed according to the required order, from the bottom to the top, successively through tension controller, positive rollers, aprons, and two wrapping areas. Fifth, the fully computerized yarn-wrapping machine started to spin after setting the appropriate spinning parameters. To obtain AIYSs with different wrapping angles, systematical twists were set at 300, 600, 900, and 1200. During fabrication, the AIYSs were collected on the groove drum-driven bobbins. After finishing the mass-productive spinning process, sixteen AIYSs with the length of approximately 20 mm were connected to the copper electrode wire using conductive silver paste, and the connection part was further encapsulated with two-component epoxy resin. Subsequently, the sensors were sewed on the selected position of a knitted glove by plain stitches. Among them, 14 AIYSs were vertically distributed on each movable joint of the five fingers, one sensor was vertically sewed in the middle of the wrist part, and the remaining sensor was horizontally connected between the index and middle fingers, as shown in Fig. 1a. According to the positions, the sensors were labeled as Thumb Top, Thumb Bottom, Index Top, Index Middle, Index Bottom, Middle Top, Middle–Middle, Middle Bottom, Ring Top, Ring Middle, Ring Bottom, Pinky Top, Pinky Middle, Pinky Bottom (sensors 1–14), Index Middle (sensor 15), and Wrist (sensor 16).

### Characterization of AIYS Performance

The morphologies of the AIYS yarns and surface morphologies of the silver-coated PA fibers were analyzed using a scanning electron microscope (TM3000, Hitachi Group, Japan) and a Dino-Lite digital microscope. After collecting the geometric pictures from the Dino-Lite digital microscope, the auxetic performance of the AIYS yarns were measured using the software ImageJ. The mechanical properties of the yarn were measured using a yarn elongation-strength tester (XL-1A, Shanghai Xinxian Instrument Co., Ltd., Shanghai, China). The testing yarn sample was clamped at the crosshead with a gauge length of 20 mm. The resistance of the AIYS was measured by using an inductance–capacitance–resistance meter (TH2829, Shenzhen Tonghui Instrument Co., Ltd., Shenzhen, China).

### Dataset Collection and Deep Learning Training Model

In terms of the training data for individual alphabet recognition, the signal data from 16 channels were recorded with 41,600 data points and 100 samples were collected for the sign language of each alphabet. Out of these 100 samples, 60 samples were randomly used for training (60%), 20 were used for validation (20%), and 20 were used for testing (20%). The volunteer wore the smart glove and repeated each letter of the alphabet (from A to Z) 100 times. To ensure data independence and the generalization ability of the dataset, two actions of full bending and full extension were interspersed between two data points corresponding to the two alphabet sign languages. The dataset was collected using a data-acquisition system (DAQ 970A, Keysight Technologies, UK). The ANN models used in the system were configured as follows: the model architecture was composed of 16 input nodes, two hidden layers with 100 nodes each, and 26 output nodes. An activation function ReLU was used for the two hidden layers. In addition, the Softmax function was used as an activation function for the output layer. The ANN was trained through backward propagation using the stochastic gradient descent method. The cross entropy loss function was used as the loss function. We periodically adjust the learning rate using a learning rate scheduler, StepLR. The learning rate decreased proportionally to 0.99 in every 50 steps of learning. 10 epochs were performed with the above conditions. The PyTorch [44] library was used for all the computations involving the ANN.

## Results and Discussion

### Design and Working Mechanism of the Sign-language Translation Glove

Figure 1a illustrates the working process of sign-language translation using the AIYS-array-embedded smart glove. The glove is fabricated by sewing a 16-element yarn sensor array on the movable joints of the fingers and wrist of a knitted glove. Firstly, the sensor array is connected with a multichannel data-acquisition system to acquire a large dataset, which is fed into the ANN algorithm to train a deep learning model. Subsequently, in real-time application, by wearing the smart glove and invoking the trained model, all the 26 letters can be translated from hand gestures into readable and audible text. As shown in Fig. 1b, the sensing unit AIYS has a unique interlocking structure, which contains two silver-coated PA yarns (sheath yarns) symmetrically wrapped on the PU yarn (core yarn).

The well-designed structure provides the AIYS with a high resistance–strain responsiveness (Fig. 1bi) and a negative Poisson’s ratio performance (Fig. 1bii) simultaneously. The auxetic structure reduces tension concentration, thereby enhancing the smart glove conformality with the human body, whereas the resistance–strain responsiveness, owing to the intrinsic slippage and elongation of the conductive sheath fiber during stretching, provides high sensitivity to the glove for different human hand movements. At the same time, it is easy to densely weave AIYS into textile gloves for sign-language recognition of the full English alphabet.

The full-fiber AIYS is fabricated using a continuous, mass-producible spinning technology with a high working efficiency and a low cost, as shown in Figs. 1c and S2 and Video S1. For the core yarn, it was firstly successively guided into the yarn-wrapping machine from bottom to top through tension controller, positive rollers, aprons, and two wrapping areas, and subsequently collected by the groove drum-driven collecting bobbins (Fig. 1c). The sets of rollers and wrap point controllers effectively control the feeding speed of the core yarn and protect them from being affected by the wrapped yarn. For the inner sheath yarn, it was fed into the first wrapping area and twisted on the surface of core PU core yarn in a clockwise winding direction to obtain a Z-twist helical structure (Fig. 1ci). For the outer sheath yarn, it is twisted in a counterclockwise winding direction on the Z-twist helical yarn surface of the second wrapping area to form an interlaced structure (Fig. 1cii). Consequently, the AIYS is continuously collected in the bobbin, as shown in Fig. 1ciii. The AIYS fabrication process has a fast working speed, and approximately 2,400 m of the AIYS can be obtained on a one-ring bobbin within 1 h (Video S1). In addition, the fabrication cost of an AIYS is very low, as calculated in Table S1 and Note S1, resulting in approximately $0.085 per meter of yarn sensor. Hence, the sign-language translation glove can be mass-produced at a low cost of less than $2.

### Geometric and Mechanical Behavior of the AIYS Sensor

Figure 2 shows that the AIYS has unique stability and a negative Poisson’s ratio performance owing to the well-designed interlaced structure. In the AIYS, two sheath yarns are wound in opposite wrapping directions and form a tight interlocked structure on the core yarn surface, as shown in Fig. 2a-b. The interlocked AIYS exhibits more stability in a tension-free state than the helical yarn sensor with only one wrapping sheath (Figs. S3 and S4) [38, 45], because the unbalanced residual torque leads to a slipping of the wrap component from the core. In addition, an evident negative Poisson’s ratio effect for the AIYS is achieved during stretching, as shown in Fig. 2b. Here, the Poisson’s ratio (ν) is the ratio of the radial contraction strain to the axial strain in the stretching force direction, that is,

1 $$\varepsilon_{r} = \frac{{d - d_{0} }}{{d_{0} }}$$

2 $$\nu = - \frac{{\varepsilon_{r} }}{{\varepsilon_{a} }}$$

where *ε*_r_ and *ε*_a_ represent the radial and axial strains of AIYS composite yarns, respectively (Fig. S4). When the AIYS is stretched from 0 to 5% elongation, the sheath yarns first wrap the core more tightly to reach the force and moment equilibria, whereas the cross-sectional area of the core yarn contracts and the diameter decreases because of the stretching. With the elongation increasing to 30%, the difference between the elastic modulus of the yarn components changes the structure of the sheath yarns from a helical wrap to straight; conversely, the core yarn changes from straight to bend, exhibiting a sinusoidal-curve shape. Consequently, the contour dimension of the AIYS rapidly increases to the maximum value and exhibits an evident auxetic performance (Figs. 2b and S5).

Owing to the unique interlaced structure, the change in the radial contour diameter of the AIYS is greater than that of the helical auxetic yarn (Fig. S5); hence, it exhibits a higher negative Poisson’s ratio performance (Figs. 2c and S5). Meanwhile, with a decrease in the initial wrapping angle (*θ*) from 69.1° to 45.7°, the geometric radial diameter changes more significantly and has a greater negative Poisson's ratio effect (Fig. 2d-e). When the initial wrapping angle is large, such as 63.1º and 69.1º, the AIYS doesn’t show an obvious negative Poisson's ratio effect; however, when it decreases to 50.9º, the AIYS shows a negative Poisson's ratio of − 0.7 at the strain 15%. The maximum Poisson’s ratio of the AIYS reaches up to − 1.5 with a wrapping angle of 45.7°. As the yarn elongates (30–50%), the diameter decreases because the compliant core straightens until the sheath yarns break. Furthermore, we theoretically calculated the Poisson’s ratio by establishing numerical models based on the geometric deformation, which are consistent with experimental results, as shown in Note S1 and Fig. S6.

The AIYS has good cycling stability (Fig. 2g) and elastic recovery within the strain range of 0–10% owing to the high elasticity of core PU yarn and the wrapped geometric morphology of the sheath yarn. Within this range, two sheath yarns are only straightened from curve to a straight state, instead of being stretched like core PU yarn. There exists a slight stress relaxation during the 1000 times repeated stretching cycling, as shown in Fig. S7. The AIYS has a breaking elongation of more than 200%, and exhibits a unique stress–strain behavior (Fig. 2f). During stretching, the axial stress increases until the state of the outer sheath yarn turn from twist to straight, and then is stretched to its breaking point (point 1 in Fig. 2f). Within this range, the AIYS is highly stable and repeatable. Subsequently, the inner sheath yarn endures the major external stresses until the axial strain reaches the next breaking point (point 2 in Fig. 2f). Then, the stress–strain curve exhibits a near elastic stretching and recovery behavior, which is similar to that of the PU yarn. Moreover, with well-aligned AIYS, a porous fabric can be formed because of the deformation of the yarn components during stretching, which is beneficial for the fabric structure design (Fig. S5). The auxetic effect of the AIYS facilitates self-expansion when an E-textile is worn on the human body, thereby resulting in better body conformality.

Figure 2h shows two theoretical viscoelasticity models established based on the interlaced core–shell yarn structure to better describe and predict the mechanical performance of the AIYS. As discussed, the AIYS is composed of one core PU yarn and two helical PA yarns with different twisting directions; therefore, it can be considered that the AIYS consists of a viscoelastic core parallel to two spring-like filaments. Considering the viscoelastic properties and interaction with the sheath yarns, a Maxwell model of a dashpot in series with a spring is used to describe the mechanical performance of the PU component. Further, two springs are used to describe the two sheath yarns; thus, a four-element model (Model-I) is established (Figs. 2h and S8a). Considering that the morphology of the inner sheath yarn is not a regular linear spring because it is subjected to the double-layer stress from the outer sheath and core yarns, a nonlinear spring is used to replace the original spring in Model-I; consequently, Model-II is established (Figs. 2h and S8b). After deriving the constitute equations based on the deformation characteristics of the basic components (Note S2), we can get the constitutive numerical models as follows:

Model-I:

3 $$\sigma = \eta k\left( {1 - e^{{ - \frac{{E_{1} }}{\eta } \cdot \left( {\frac{\varepsilon }{k}} \right)^{p} }} } \right) + E_{1} \varepsilon + E_{2} \varepsilon$$

Model-II:

4 $$\sigma = \eta k\left( {1 - e^{{ - \frac{{E_{1} }}{\eta } \cdot \left( {\frac{\varepsilon }{k}} \right)^{p} }} } \right) + C\varepsilon^{n} + E_{2} \varepsilon$$

where *η* is the viscosity coefficient of the ideal dashpot, *E*_1_ and *E*_2_ represent Young’s modulus of two spring elements in the designed models, $\varepsilon$ and $\sigma$ are the strain and stress of AIYS, *p* is the function correction factor. Then, we fitted the experimental data with our proposed numerical models using the Origin software. The fitting aptness of the constitutive models is evaluated based on widely accepted statistical criteria, such as the determination coefficient (*R*^2^).

A determination coefficient of 0.999 is observed for the theoretically and experimentally derived stress–strain curves (Figs. 2h and S8) using Model-II, whereas only 0.992 is achieved by Model-I. Because it considers the inner sheath yarn tension and has a nonlinear mechanical behavior, Model-II is more consistent with the viscoelastic behavior of AIYS than Model-I. The results show that the predictions from Model-II are in closer agreement with the measured mechanical performances in the strain range, thus demonstrating that Model-II is more suitable for analyzing and characterizing the mechanical properties of the AIYS. The new mechanical constitutive model, which fully considers the structural distribution and nonlinear mechanical behavior of the AIYS, is of significant value to better understand the mechanical behavior of the intelligent yarn sensor and provide guidance for the parameter design of the E-textiles.

### Sensing Performance of the AIYS Sensor

**Fig. 3:**
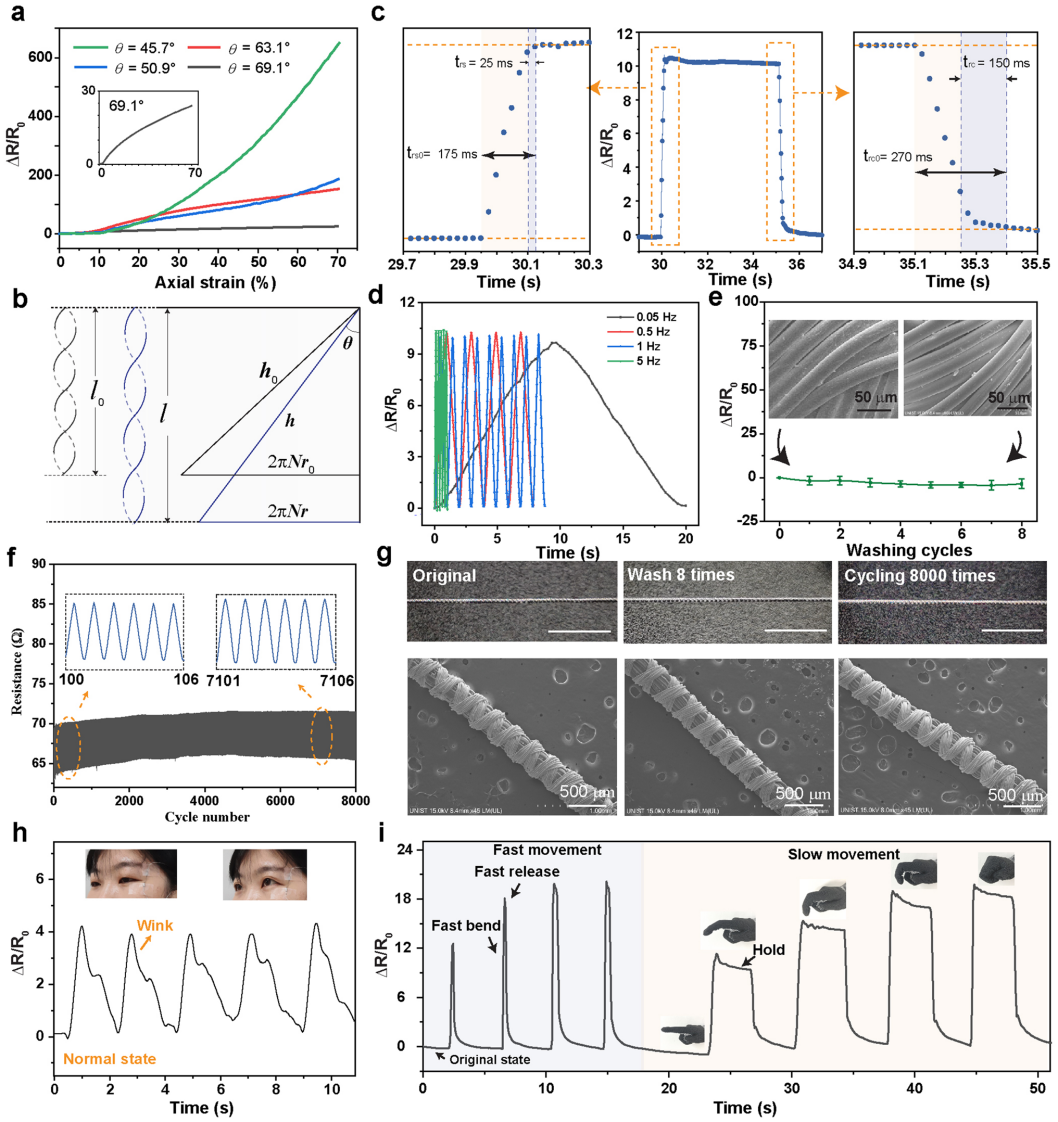
Sensing performance of the AIYS sensing. **a** Relative change in resistance (Δ*R*/*R*_0_) of AIYS during stretching. **b** Response and recovery time (*t*_rs_ and *t*_rc_) of the AIYS sensor when stretched to 15% strain. **c** Illustration of the unfolding of the spiral sheath yarn. **d** Response of AIYS under the frequency range of 0.05–5 Hz. **e** Resistance variation of the AIYS after continuous washing test. Insets depict the SEM images of the conductive part of AIYS, showing no obvious change. **f** Cyclic electrical performance of AIYS sensor for stretching and recovery 8000 times; insets depict the amplification of the signal near the beginning (Cycle No. 100–106) and the end of the test (Cycle No. 7101–7106). **g** Optical and SEM images of the AIYS in the states of origin with washing 8 times and cycling 8,000 times. Scale bar of 50 mm. **h** Detected signals of human winking when volunteers wear the AIYS at the eye corner, the insets are the pictures showing the upper facial expressions around the eyes. **i** Real-time sensing performance of the AIYS-embedded glove after fast bending, slow bending, keeping, and releasing behaviors

Figure 3a shows that the AIYS exhibits a good performance in strain sensing, with significant variation in resistance during stretching. The sensing mechanism relies on the contact resistance between the sheath yarn spiral units and the squeezing of the fiber bundles during stretching. According to the geometric structure of the conductive PA sheath filaments are bundled together, wrapping on the PU core fibers with a certain angle *θ*. Considering that PA and PU are insulating materials with a higher electrical resistance than conductive silver, their conductivities are ignored in the AIYS. The equivalent resistance of a wrapping unit of the AIYS can be regarded as two yarn sheath length resistances (*R*_i1_ and *R*_i2_) in parallel and connected with a contact resistance (*R*_i3_) in between. During stretching or bending, the state of the wrapping conductive yarn changes from the helical wrap to straight, and subsequently the yarn continues to be stretched until it breaks (Fig. 2b). If the helical yarn is stretched along the central axis (Fig. 3b), the increase in the pitch (*h*) decreases the radius (*r*) and increases the length (*l*) of the sheath yarn, which simultaneously leads to an increase in the length resistance (*R*_i1_, *R*_i2_). Moreover, during the stretching of the AIYS, the contact area between the two sheath yarn layers decreases, which also results in an increase in the contact resistance (*R*_i3_) between the sheath fibers. When the initial wrapping angle is very high, parts of the sheath wrapping fibers are initially connected with each other because of the decreasing of pitch distance; hence, gaps are generated between the helical units during stretching, increasing the overall resistance of the AISY. The AIYS with a smaller wrapping angle shows better responsiveness under the axial strain, which is attributed to its significant geometric deformation and auxetic effect during stretching.

Figure 3c shows a response time of 25 ms (*t*_rs_) while loading and unloading a 15% strain to the sensor with high speed and holding it for 5 s. The *t*_rs_ (*t*_rc_) is defined as *t*_rs_ (*t*_rc_) = *t*_rs0_ (*t*_rc0_) – *t*_0_, where *t*_rs0_ (*t*_rc0_) is the measured response or recovery time and *t*_0_ denotes the time required for strain loading or unloading [24]. More tests on the loading in different strains (0–20%) shows that the response and recovery timed of AIYS are less than 50 and 150 ms, respectively (Fig. S9). This phenomenon is attributed to the superior resilience of the core PU yarn. Moreover, the fast response of the AIYS can be verified by the stable response under a high stretching frequency of 5 Hz (Fig. S10). As shown in Fig. 3d, the AIYS has a similar response and good cycling performance under a mechanical frequency of 5 Hz with a relatively low frequency of 1, 0.5, and 0.05 Hz. Figure 3c-d also show the good resistance recoverability of the AIYS when the stress is released, which is due to the high elasticity of core PU yarn and wrapped geometric morphology of the sheath yarn. In addition, we tested the washability of the conductive yarn and AIYS with detergent and water in a beaker. As shown in Figs. 3e and S11, AIYS does not show an obvious change in morphology for the eight times that it is washed, and the electroplated silver layer is still uniform on the surface. Therefore, the resistance does not show an obvious fluctuation. The slight decrease in resistance is caused by the loosening of the fiber bundle during washing, as seen from the optical images in Fig. S9c. The AIYS also shows an excellent cyclic stability during the 8,000 times that it is stretched and released, as shown in Fig. 3f. The slight upshift in the baseline during the cycling is caused by the stress relaxation of the AIYS. The amplification of the signal in cycle numbers 101 and 7101 shows that there are continuous stable responses under repeated stretching. The optical and SEM images in Fig. 3g show that the AIYS maintains its interlaced structures under repeated washing and cyclic stretching tests. The washed AIYS shows an unchanged performance as compared to that of the original AIYS (Fig. S12). Meanwhile, after the AIYS is stretched for 8,000 times, it shows a slightly decreased signal owing to the polymer stress relaxation. Because of the fast response and good sensitivity of AIYS, it can be utilized for human facial expression detection and translation. When the AIYS is worn on human skin, a small movement signal of winking or coughing can be detected, as shown in Figs. 3h and S13. Furthermore, the AIYS is attached to the index-finger joint part of the knitted glove, which generates unique signals to different joint bending information such as fast and slow movements, bending, releasing, and holding, as shown in Fig. 3i.

### Smart Glove for Full letter Sign-language Recognition and Real-time Dialogue Translation

**Fig. 4:**
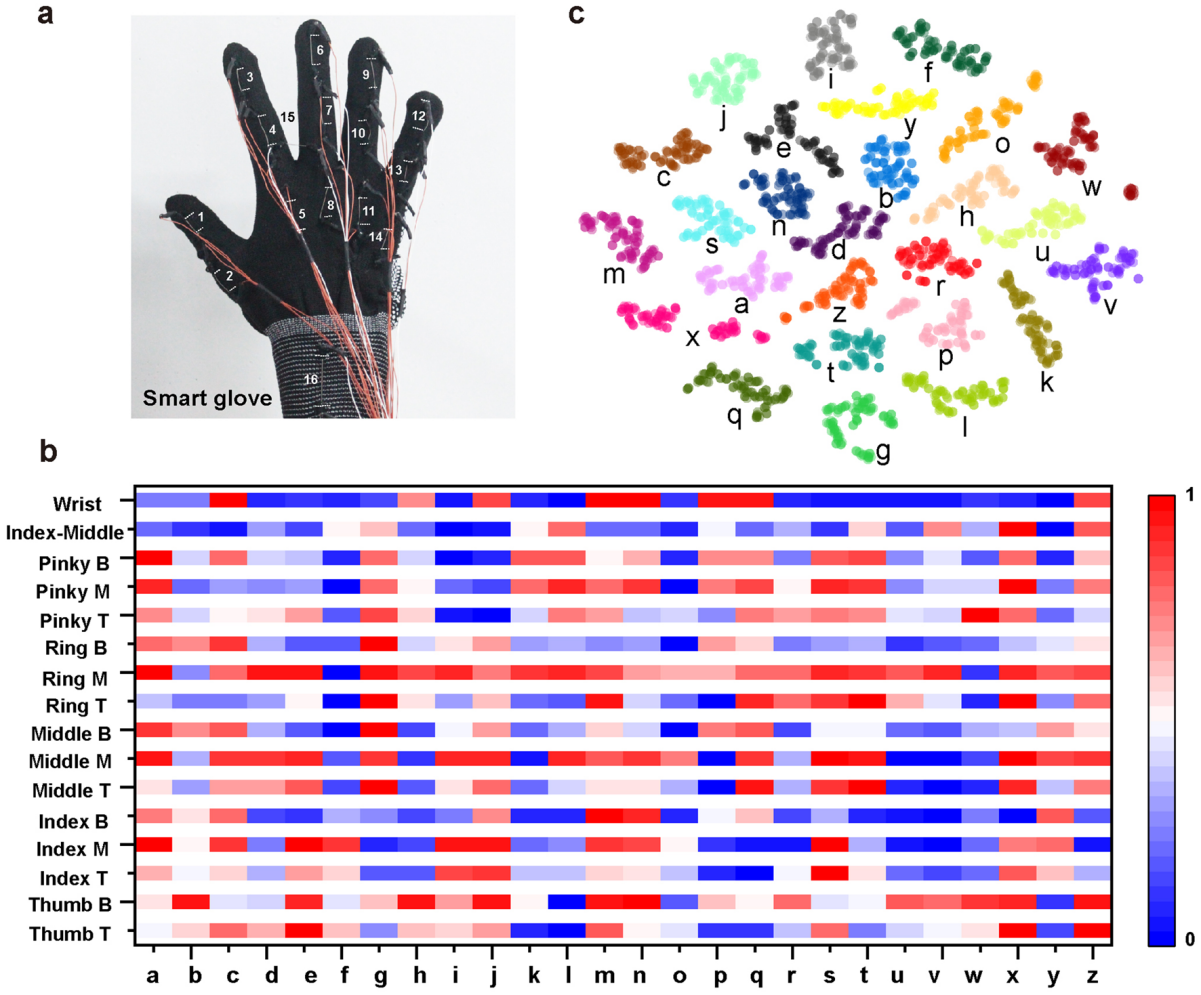
Working mechanism of the sign-language translation glove. **a** Photograph of a smart glove embedded with 16 channel sensors. **b** Signal matrix for sensor-bending situation when the smart glove makes the sign-language gestures from “a” to “z.” 0” indicates no bending or stretching, and “1″ indicates full bending or stretching. **c** *t*-SNE plot of alphabet signal dataset recorded by the glove enabled with 16 AIYS array

Figure 4 shows the working mechanism of the knitted glove embedded with 16 AIYSs for distinguishing the different signs of the 26 letters of the alphabet. Among them, 14 AIYSs are vertically distributed on the movable joints of five fingers, one AIYS is horizontally connected between the index and middle fingers, and the remaining sensor is vertically sewn in the middle of the wrist part, as shown in Fig. 4a. According to the sign-language gestures based on ASL (Fig. S14), most of the signs for the letters of the alphabet have apparent differences in bending situation and can be distinguished by the sensors distributed on the back of the joint part (sensors 1–14), except for some similar letters such as “u” and “v,” and “k” and “p,” which require two additional sensitive sensors with a specialized purpose (sensors 15 and 16). For the letters whose sign languages contain a motion (“j” and “z”), the last signal of the movement is taken as its training data. The electrical signals from all AIYSs are captured using a data-acquisition system, as illustrated in Fig. S15. After data normalization for each sensor, the bending and stretching situations of each alphabet were counted and analyzed, as shown in Fig. 4b. The color bar exhibits the degree of movement of each joint part, where blue indicates no bending or stretching, and red indicates full bending or stretching. As shown in Figs. 4b and S16, most of the letters have a distinguishable combination of bending situations detected by the 14 finger joint sensors. The rest of them, such as “u” and “v” can be distinguished by sensor 15 (Index-Middle), and “k” as “p” can be distinguished with the help of sensor 16 (Wrist). Therefore, each letter (from A to Z) shows a different combination of bending or stretching situations between the sensors. Meanwhile, it is shown that among all the joint movements contributing toward the sign language, the ring and pinky fingers have frequent bending movements than the other three fingers.

During the repeated bending and recovery, we noticed that the sensors encountered the problems of baseline shift, stress relaxation, and position movement, which can be also seen from Figs. 3f and S10. To overcome these problems, we further used the ANN algorithm for the sensor calibration and correction, and letter sign-language signal classification. We firstly prepared a dataset comprising 2,600 data points, that is, 100 data points for each letter of the alphabet. Subsequently, a t-distributed stochastic neighbor embedding (*t*-SNE) plot [46], which is a dimensionality reduction technique to visualize the group of datasets was generated, as shown in Fig. 4c. Each point on the plot represents one gesture information projected from the sensor data. The data points that belong to the same letter category are clustered together, roughly generating 26 categories. There is no evident overlapping between the dataset, indicating the distinguishability of the 26 signs.

**Fig. 5:**
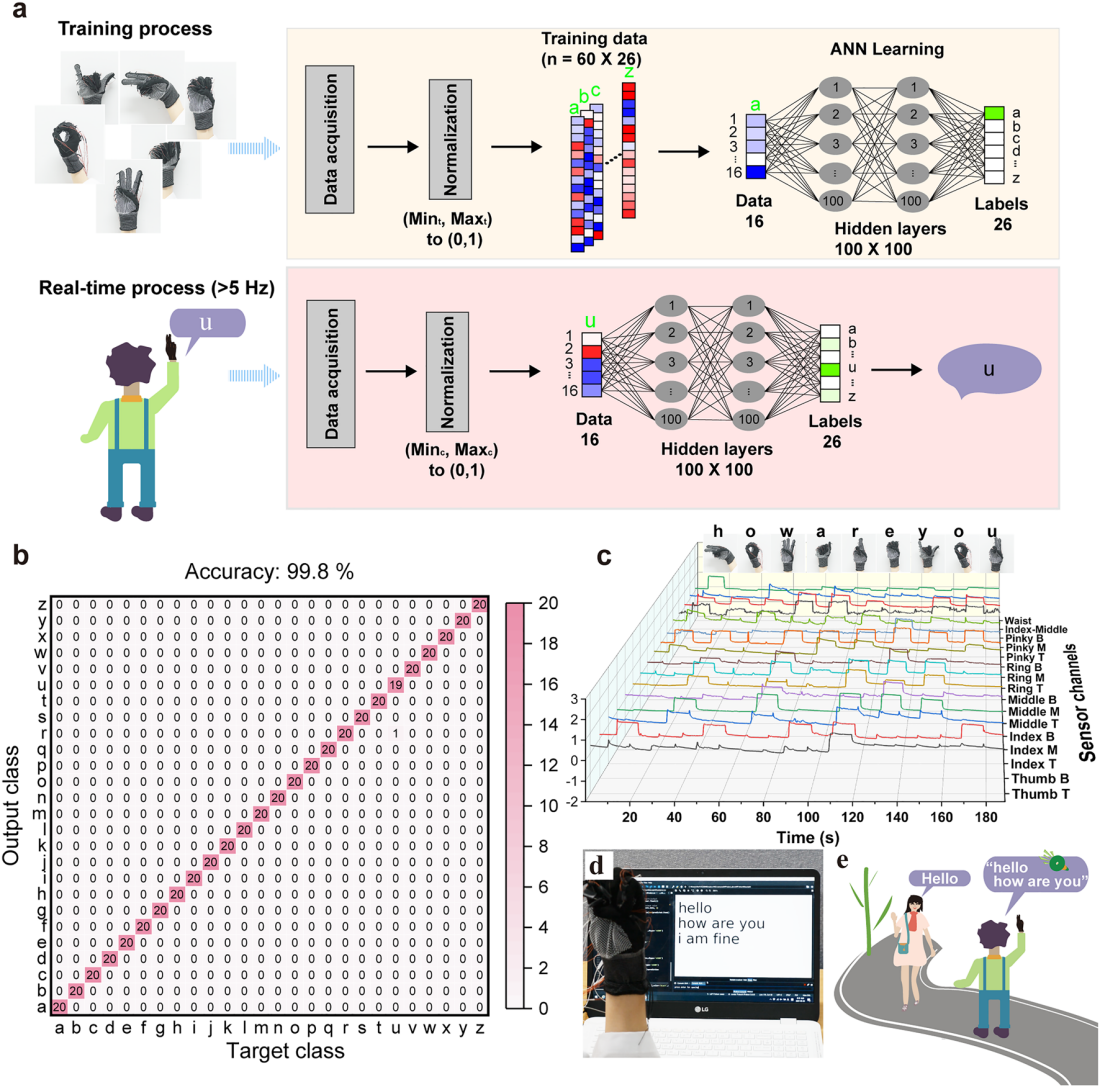
Deep-learning-enabled all-alphabet sign recognition and real-time dialogue translation. **a** Training and real-time process of 26-letter-recognition and translation system, and detailed ANN architecture. **b** Confusion matrix for individual recognition of all letters, exhibiting a high overall accuracy of 99.8%. **c** Collected real-time-normalized data when a continuous dialogue sign “how are you” is demonstrated. **d** Experiment showing the input sentence in real time from the sign language demonstrated by the volunteer wearing the smart glove. **e** Illustration showing a signer communicating with a person with the help of the sign-language translation glove

Figure 5a illustrates the detailed process of sign-language classification using the ANN architecture. The multichannel resistance signals of the AIYS array were fed into the deep learning algorithm after normalization. The sensor signals acquired from each volunteer were normalized by the minimum (Min_t_) and maximum (Max_t_) signals of the individuals. Subsequently, a total of 1,560 data points (60% of the dataset) were randomly selected from the acquired signals to serve as the training set, and 520 data points (20% of the dataset) were used as the validation set. The remaining 520 data points (20% of the dataset) were used as the test set. The training set was used to train the ANN, which consisted of two hidden layers with 100 nodes in each layer. Thereafter, using the trained ANN, we built the real-time sign-language classification model that caters to a frequency greater than 5 Hz (which is the frequency of our data-acquisition device). The confusion matrix of the classification result is presented in Fig. 5b. Each column of the matrix represents the test samples in an actual class, while each row represents a predicted class. The results demonstrate that the sign signals for 25 letters achieved a classification accuracy of 100%, whereas the one remaining letter “u” achieved an accuracy of 95%, because of the gesture similarities between “u” and “r.” The overall accuracy is 99.8% and the average recognition time for the entire gesture class is less than 0.25 s.

More importantly, our all-alphabet recognition glove can be used as a movable and wearable keyboard to freely input and translate complex sentences and common dialogues into text or voice in real-time. This cannot be fulfilled by other sign-language translation systems with limited ability for alphabet recognition [14]. On wearing the smart glove, a volunteer can make the sign-language gestures from “A” to “Z” by invoking the established deep learning model, and the corresponding letters can be immediately translated into text, as shown in Fig. S17 and Videos S2 and S3. Based on this, the smart glove also output the voices or texts of sentences by inputting the signs of the 26 letters in sequence. For example, when the volunteer wore the smart glove and made the alphabet letter signs for the sentences they wanted to express in sequence, such as “Hello,” “How are you,” “I am fine,” and other complex dialogues (Figs. S18 and S19), they were transferred to texts without any apparent delay (Fig. 5d and Video S4). Figure 5c shows the normalized data collected from the sentence “How are you.” Here, the breaks between the words were input manually using a pre-set key to show the results more clearly.

As illustrated in Fig. 5e, it is worthy to further compare our sensor-based sign-language translation glove with a vision-based sign-language translation system. As reported, the latter has a drawback of various challenges faced during video/image processing such as lighting conditions, brightness, background noise, and camera angle [21]. In contrast, the former has a strong ability that is not affected by the users’ environment and allows wearers to move freely while using it. In addition, our smart glove not only has more advantages such high portability, low-cost (< $2), and high recognition accuracy, it can also translate sign-language owing to its ability to recognize and translate all letters. Furthermore, our smart glove can be easily integrated with various portable devices such as cellular phones or smart watches, making it easy for the ANN algorithm developed in this work to be implemented as a mobile app, which can translate the sign language in real-time into text messages, voices, and braille-writers without the limitations of locations. Therefore, our smart glove could provide a new light for eliminating the existing associated barriers that hinder the communication between signers and non-signers.

## Conclusions

In this work, a sign-language translation glove is developed using auxetic-interlaced AIYS array and a deep learning algorithm. The AIYS was fabricated using a continuous and mass-producible interlaced yarn-wrapping technology at a high speed and low cost. The prepared AIYS sensing unit exhibited a well-stabilized geometric structure, high negative Poisson’s ratio performance (− 1.5), excellent mechanical–electrical performance, high strain sensitivity, fast response (0.025 s), and sufficient repeatability and reliability (> 8,000). In addition, we established four-element viscoelastic models that theoretically consider the nonlinear elastic behavior of sheath yarns in a comprehensive manner. The theoretical models, which describe the mechanical behavior of the AIYS, not only showed consistency with the experimental results (*R* = 0.999) but also made it possible to engineer the excellent mechanical–electrical performance. Moreover, we demonstrated that the smart glove sewn with 16 channels of the AIYS can completely recognize all the signs of the 26 letters of the alphabet by processing the multichannel-collected resistance data with deep learning algorithm. Using the ANN algorithm, we successfully classified 2,600 sign-language gestures covering the 26 letters, and obtained a high recognition accuracy of 99.8% for all 26 letters with a short recognition time (< 0.25 s). Thus, we demonstrated that the smart glove allows sign language to be real-time translated into text or voice, which can eliminate the communication barriers of signers in a portable, convenient, simple, and inexpensive manner.

## Supplementary Information

Below is the link to the electronic supplementary material.

Supplementary file1 (AVI 3173 KB)

Supplementary file2 (AVI 16004 KB)

Supplementary file3 (AVI 7442 KB)

Supplementary file4 (AVI 11797 KB)

Supplementary file5 (PDF 2473 KB)

## Declarations

### Data availability

The data that support the plots within this paper and other findings of the study are available from the corresponding author upon reasonable request.

### Code availability

The code is available from the corresponding author upon reasonable request.
