# Supplementary material for: Full-Fiber Auxetic-Interlaced Yarn Sensor for Sign-Language Translation Glove Assisted by Artificial Neural Network
Source: Nanomicro Lett. 2022 Jul 1;14:139. doi: 10.1007/s40820-022-00887-5 (PMC9249965; doi:10.1007/s40820-022-00887-5)
Supplement: Supplementary file 5 — Supplementary file5 (PDF 2473 KB) [file 40820_2022_887_MOESM5_ESM.pdf]

Supporting Information for

Full-Fiber Auxetic-Interlaced Yarn Sensor for Sign-Language Translation Glove Assisted by Artificial Neural Network

Ronghui Wu[1, †], Sangjin Seo[1 †], Liyun Ma[2], Juyeol Bae[1], and Taesung Kim[1, *]

[1]Department of Mechanical Engineering, Ulsan National Institute of Science and Technology (UNIST), 50 UNIST-gil, Ulsan 44919, Republic of Korea

[2]College of Physical Science and Technology, Xiamen University, Xiamen 361005, P. R. China

†Ronghui Wu and Sangjin Seo contributed equally to this work

*Corresponding author. E-mail: tskim@unist.ac.kr (Taesung Kim)

S1 Supplementary Tables

Table S1 Price of raw materials for the AIYS sensor and smart glove

Item	Parameter	Unit Price
PA conductive yarn	200 D	\$1.42/100 m
PU yarn	800 D	\$0.05/100 m
Knitted glove	Ribbed weave structure	\$1/pair

Note that, for the interlaced wrapping yarn with a wrapping angle of 69.1°, the length of the PA yarn (L) to prepare one meter of the AIYS sensor is 5.56 m, according to the following formula (i.e., $L = 2l/cos\theta$, where l is the length of the AIYS yarn, θ is the wrapping angle, and L is the length of the required wrapping yarn. According to the price of the raw materials shown in Table S1, the cost for a one-meter-long AIYS sensor with the wrapping angle of 69.1° is approximately \$0.085. Therefore, for one smart glove with 16 AIYSs, the total cost of raw materials is less than \$2.

S2 Supplementary Notes

Note S1: Theoretical analysis of negative Poisson's ratio

In the initial state, the diameter of the AIYS composite yarn is

$$d_0 = 2(R_0 + 2r_0) \tag{S1}$$

where R_0 and r_0 are the radii of core and sheath yarns, respectively. When the AIYS is stretched to strain ε_a, the helical angle of the sheath yarn decreases from initial angle θ_0 to θ as shown in Fig. S6. The sheath yarn has no extension and obeys the helical path, while the helical distance of one unit helix increases from initial length h_0 to h_0 (1+ε_a). Then, the helical angle of the sheath yarn can be derived as:

$$cos\,\theta = \frac{h_0(1+\varepsilon_a)}{l_0} \tag{S2}$$

where ε_a is the axial strain of AIYS. As shown in Fig. S1,

$$l_0 = \frac{h_0}{cos\,\theta_0} \tag{S3}$$

Hence, we can know the helical angle during stretching as:

$$\theta = \arccos[(1+\varepsilon_a)\cos\theta_0] \quad \text{(S4)}$$

According to the assumption, the core filament deforms according to the helical path of the wrap filament. The helical radius of core yarn (w), which means the distance between the two center lines of core yarn and the auxetic yarn, can be obtained as follows:

$$w = (R + r_0) - \frac{l_0 \sin\theta}{2\pi} \quad \text{(S5)}$$

where R is the radius of the deformed core filament. It can be obtained from the following equation:

$$R = (1 - \sigma_c \varepsilon_a) R_0 \quad \text{(S6)}$$

where v_c is the Poisson's ratio of the PU core yarn. Then, we have

$$w = (1 - v_c \varepsilon_a) R_0 + r_0 - \frac{l_0 \sin\theta}{2\pi} \quad \text{(S7)}$$

The AIYS yarn diameter after stretching can be calculated as follows:

$$d = 2(R + w) = 4(1 - v_c \varepsilon_a) R_0 + 2r_0 - \frac{l_0 \sin\theta}{2\pi} \quad \text{(S8)}$$

By combining these equations with the definition of Poisson's ratio, we have

$$v = -\frac{4\pi R_0 - 8\pi R_0 v_c \varepsilon_a - l_0 \sin\theta}{4\pi\varepsilon_a (R_0 + 2r_0)} \quad \text{(S9)}$$

As shown in the equation, when the helical radius w is greater than the diameter of the sheath yarn ($2r_0$) and the diameter change of the core yarn, the AIYS diameter (d) exceeds its initial diameter (d_0), which results in a biaxial extension and a negative Poisson's ratio. In our yarn, the diameter of the core yarn is five times that of the sheath yarn, that is, $R_0=5r_0$, and the Poisson's ratio of the PU core yarn (v_c) is near 0.475 [S1, S2]. When the yarn strain reaches 30%, the wrapping angle θ become 0. Under these conditions, we can obtain the theoretical Poisson's ratio of AIYS when it is stretched to 30%: v = -1.19, which is close to the experimental result (-1.18).

Supplementary Note S2

The constitutive equation of the Model-I and II can be derived as follows:

Because of the deformation characteristics of the Maxwell model, the total strain of Maxwell model is the sum of the spring 1 and dashpot 1:

$$\varepsilon = \varepsilon_1 + \varepsilon_2 \quad \text{(S10)}$$

The total stress for the Model- II is the sum of the partial stresses of three components

$$\sigma = \sigma_1 + \sigma_2 + \sigma_3 \quad \text{(S11)}$$

Each stress component can be expressed by the corresponding strain as follows:

$$\sigma_1 = E_1\varepsilon_1 = \eta\frac{d\varepsilon_2}{dt} \tag{S12}$$

$$\sigma_2 = C\varepsilon^n \ (n > 0) \tag{S13}$$

$$\sigma_3 = E_2\varepsilon \tag{S14}$$

Based on the above relations and the deformation principle of the spring and the sticky pot under tension, the differential relation of the constitutive model is derived.

$$\frac{d\varepsilon}{dt} = \frac{d\varepsilon_1}{dt} + \frac{d\varepsilon_2}{dt} = \frac{1}{E_1}\frac{d\sigma_1}{dt} + \frac{\sigma_1}{\eta}$$

$$= \frac{1}{E_1}\left(\frac{d\sigma}{dt} - nC\varepsilon^{n-1}\frac{d\varepsilon}{dt} - E_2\frac{d\varepsilon}{dt}\right) + \frac{1}{\eta_1}(\sigma - C\varepsilon^n - E_2\varepsilon) \tag{S15}$$

This experiment was carried out under the constant-speed stretching mode, we have:

$$\frac{d\varepsilon}{dt} = k \quad or \quad \varepsilon = kt \tag{S16}$$

The experiment was carried out under the constant-speed tension mode, and the formula (S7) was substituted for (S13). The stress-strain constitutive equation including the model- II was formulated as follows:

$$\frac{d\sigma}{dt} + \frac{E_1}{\eta_1}\sigma = (E_1 + nC\varepsilon^{n-1} + E_2)\frac{d\varepsilon}{dt} + \frac{E_1E_2}{\eta}\varepsilon + \frac{CE_1}{\eta}\varepsilon^n$$

$$= k^n\frac{CE_1}{\eta}t^n + k^n nCt^{n-1} + k\frac{E_1E_2}{\eta}t + E_1k + E_2k \tag{S17}$$

Based on the mathematical method, we have

$$\sigma = e^{-\int\frac{E_1}{\eta}dt}\left[\iint\left(k^n\frac{CE_1}{\eta}t^n + k^n nCt^{n-1} + k\frac{E_1E_2}{\eta}t + E_1k + E_2k\right)e^{\int\frac{E_1}{\eta}dt}dt + C_0\right]$$

$$= C_0e^{-\int\frac{E_1}{\eta}dt} + e^{-\int\frac{E_1}{\eta}dt}\left(Ck^nt^ne^{\frac{E_1}{\eta}t} + E_2kte^{\frac{E_1}{\eta}t} + \eta ke^{\frac{E_1}{\eta}t}\right)$$

$$= C_0e^{-\frac{E_1}{\eta_1}t} + Ck^nt^n + E_2kt + \eta k \tag{S18}$$

The stress-strain relationship of can be obtained according to the initial condition σ（t=0）=0:

$$\sigma = \eta k\left(1 - e^{-\frac{E_1}{\eta}\cdot\frac{\varepsilon}{k}}\right) + C\varepsilon^n + E_2\varepsilon \tag{S19}$$

Furtherly, to enable the theatrical model more close to the experimental result, we introduce an exponential function correction factor ($p > 0$) [S3-S5], therefore, the constitute equation was derivate as follows:

Model-II：
$$\sigma = \eta k\left(1 - e^{-\frac{E_1}{\eta}\cdot\left(\frac{\varepsilon}{k}\right)^p}\right) + C\varepsilon^n + E_2\varepsilon \quad \text{(S20)}$$

For model-I, the constitute equation is same as model-II when the spring is linear (n=1). Therefore, the constitute equation is as follows:

Model-I：
$$\sigma = \eta k\left(1 - e^{-\frac{E_1}{\eta}\cdot\left(\frac{\varepsilon}{k}\right)^p}\right) + E_1\varepsilon + E_2\varepsilon \quad \text{(S21)}$$

Note: η represent the viscosity coefficient of ideal dashpot, and E_1 and E_2 represent the Young's modulus of two ideal spring elements in the designed models, which are considered constant during stretching.

S3 Supplementary Figures

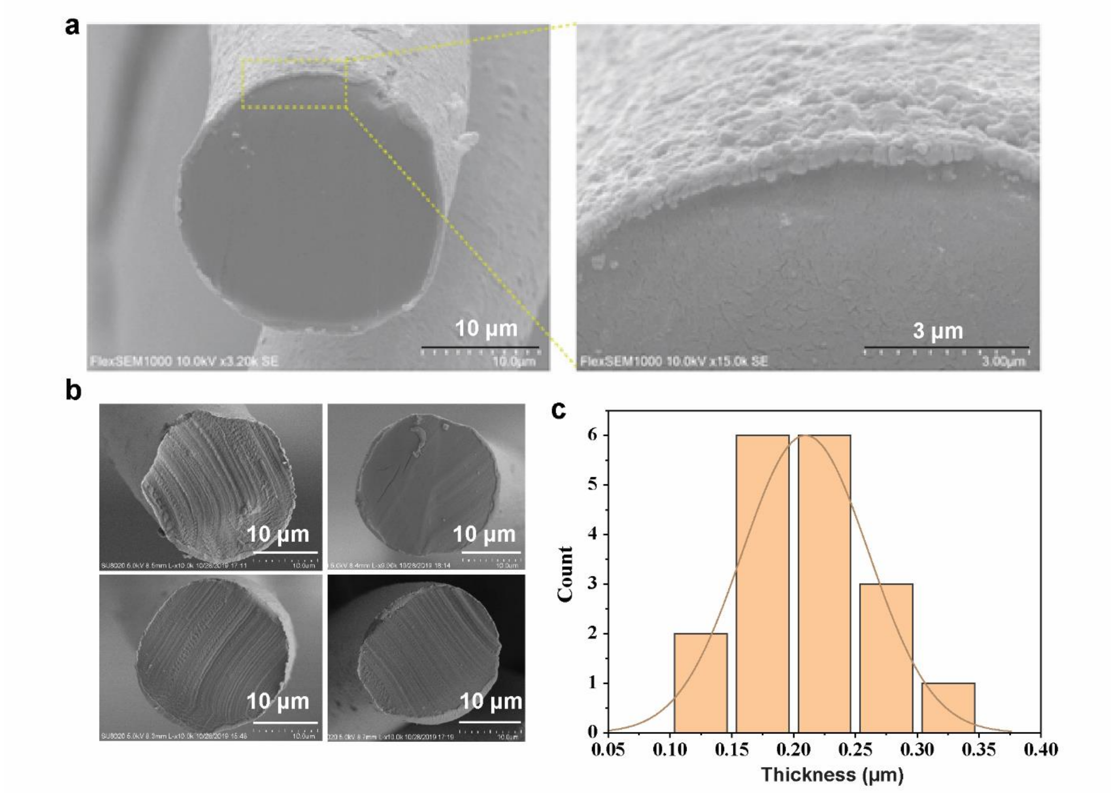

Fig. S1 Morphology of the sheath silver-coated PA filament. (**a**) Cross-sectional SEM image and its partial magnification of the silver-coated PA filament. (**b**) Cross-sectional SEM images showing the uniformity of silver coating on the PA filament surface. (**c**) Statistical distribution graph of the thickness of the silver coating on the PA fiber surface

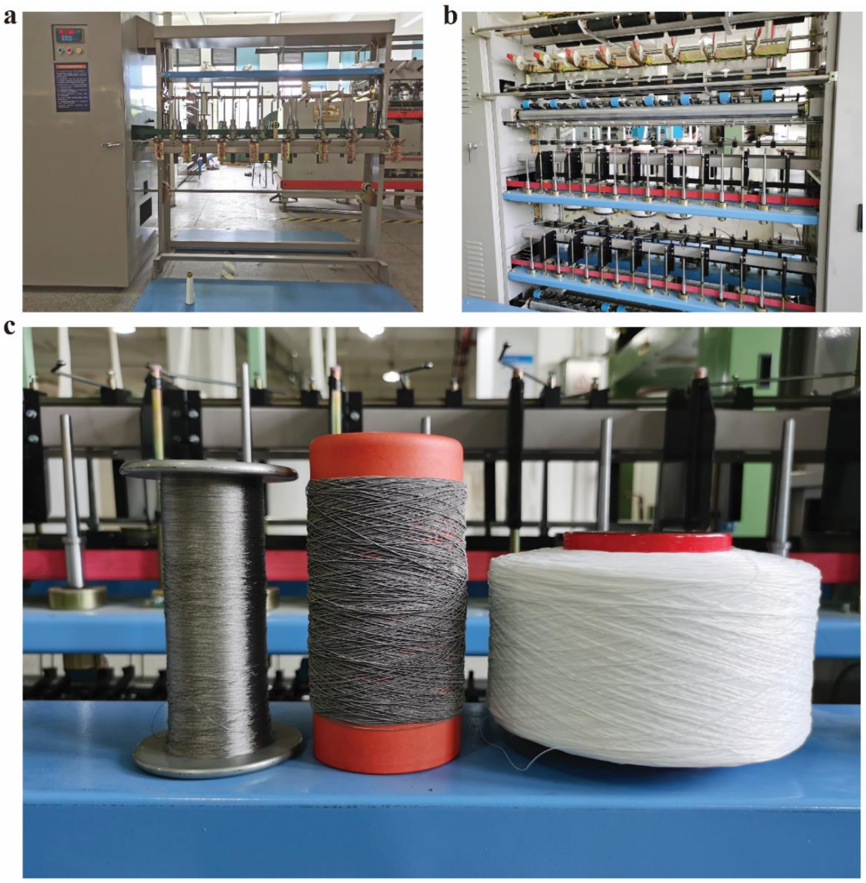

Fig. S2 Pictures showing the continuous and mass-producible AIYS spinning technology. (**a**) Yarn pressing machine, which is used to prepare the bobbin of wrapping yarn. (**b**) Hollow spindle twister machine for fabricating the AIYS. (**c**) Pictures show the core yarn (right), sheath yarn (left), and AIYS yarn (middle)

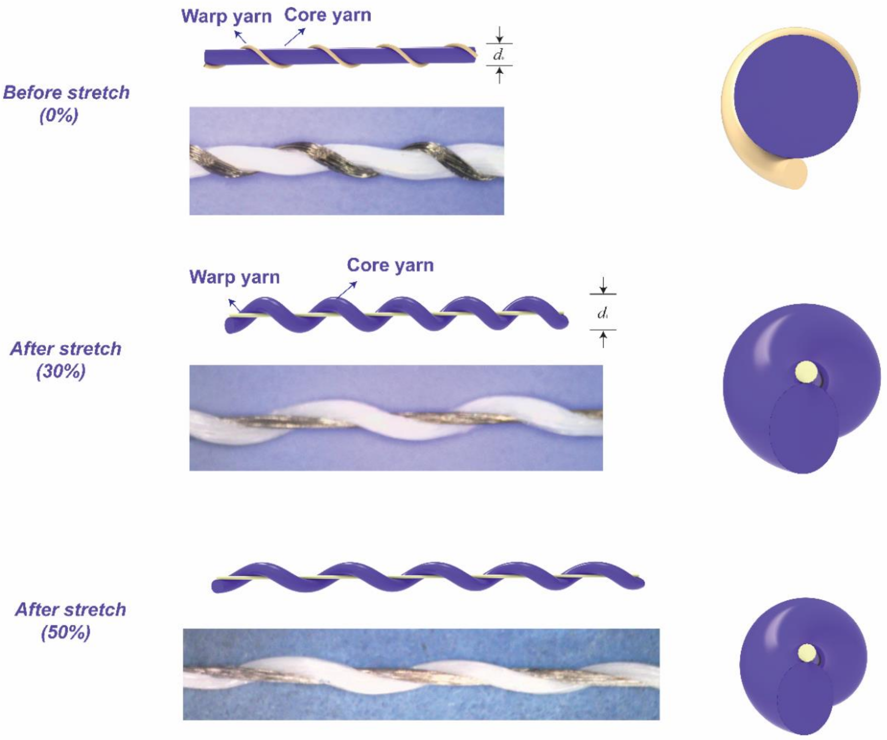

Fig. S3 Different states when the helical auxetic yarn is elongated from 0% to 30% and 50%

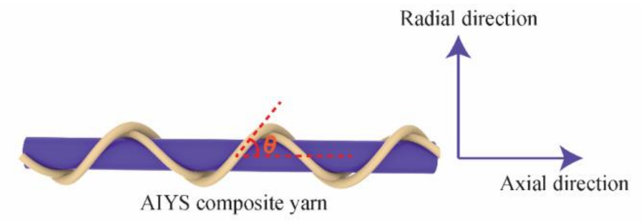

Fig. S4 The axial and radial direction of the AIYS composite yarn

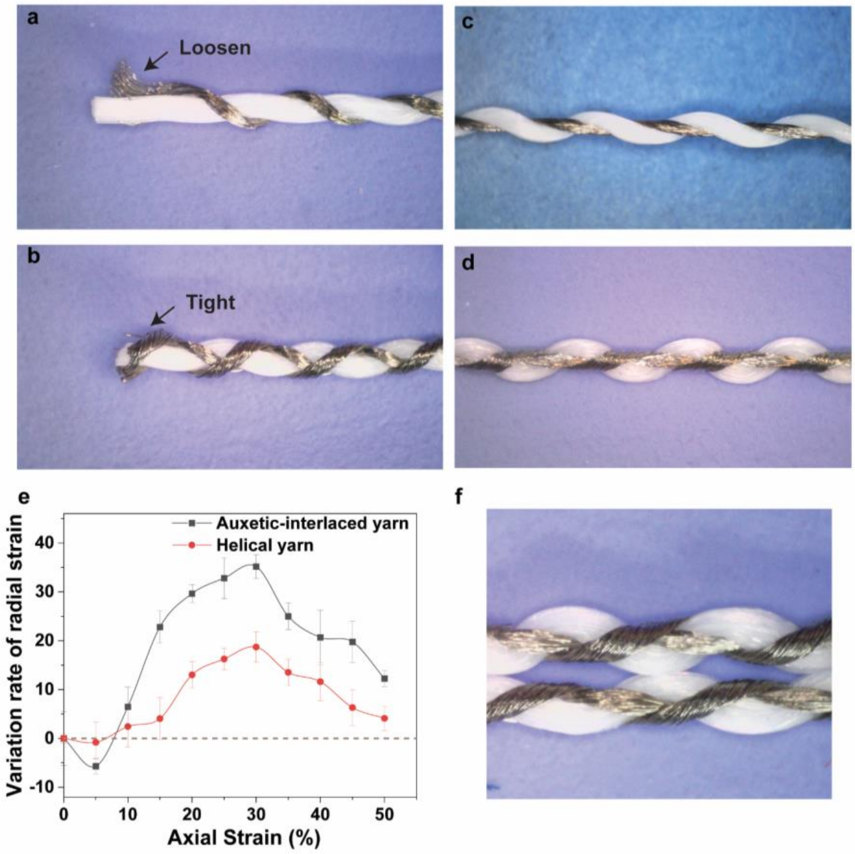

Fig. S5 (**a-b**) Natural state of the helical and interlaced helical yarn in the absence of tension. (**c-d**) Morphology of the helical and interlaced helical yarn in the stretched state. (**e**) Variation in the radial strains of interlaced-helical and single-helical auxetic yarn during stretching. (**f**) Mesh effect exhibited by a complementary pair of the AIYS in a stretched state

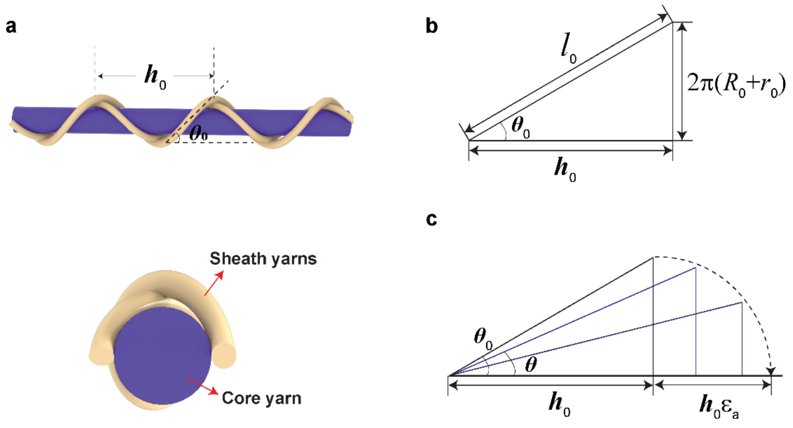

Fig. S6 The structural models the AIYS yarn. (**a**) Structure illustration of AIYS yarn. (**b**) Expanded graph of the helical unit of one wrapping filament. (**c**) Expanded graph of the wrapping filament in different states when AIYS is stretched

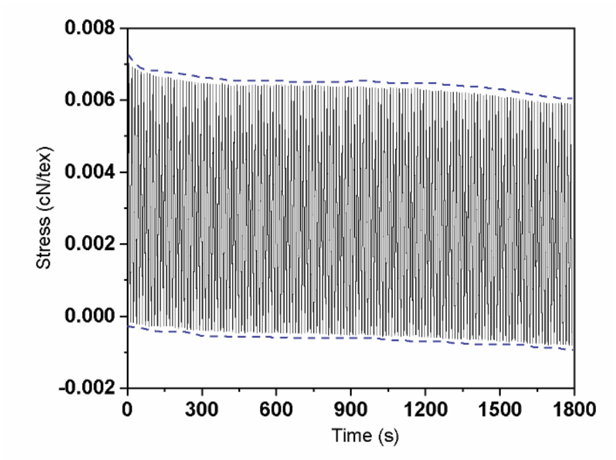

Fig. S7 Recycling mechanical test (1000 times) for the AISY sensor

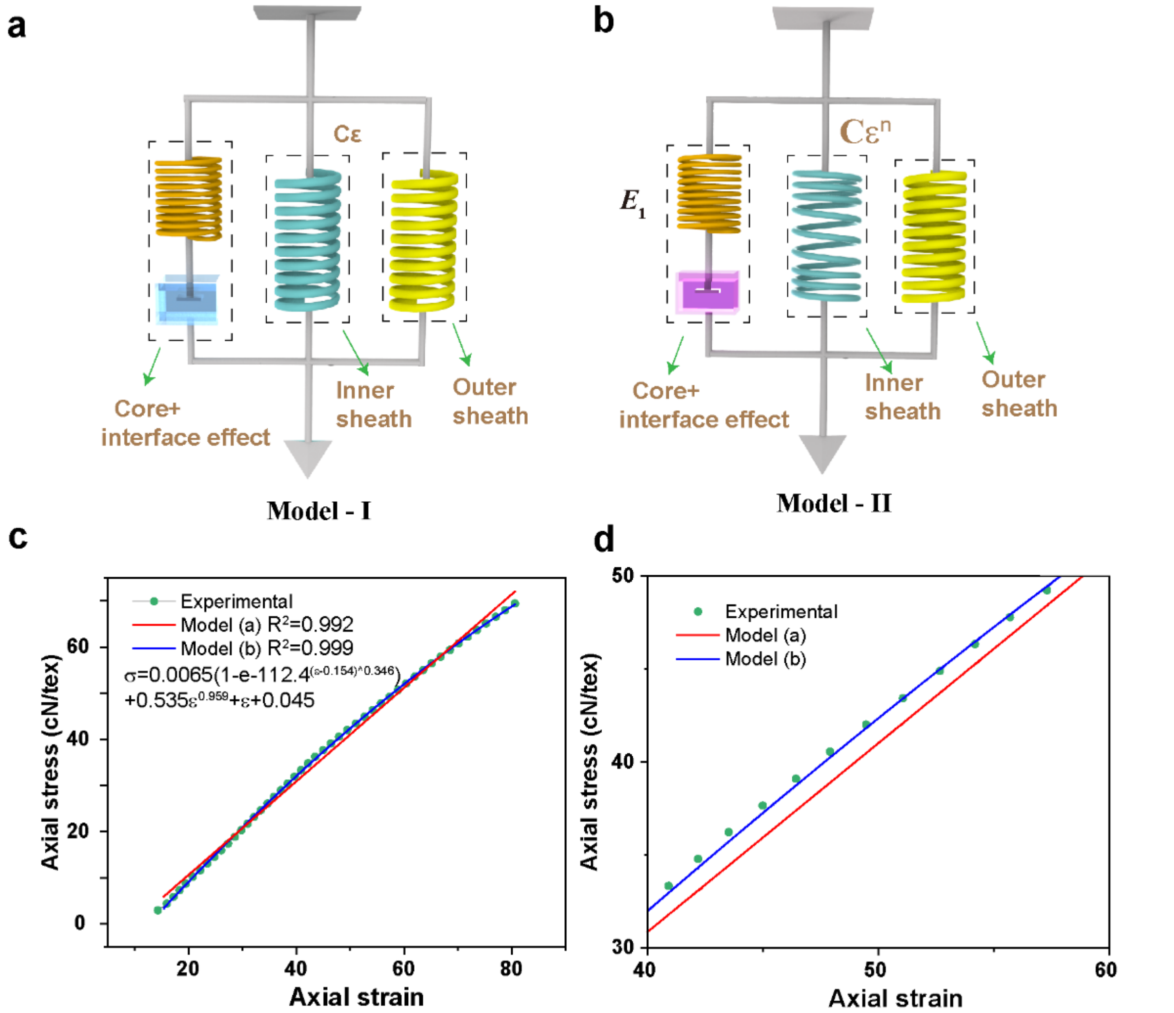

Fig. S8 Theoretical models for AIYSs. (**a**) Illustration of Model-I, where a spring in series with a dashpot is used to describe the mechanical behavior of the core yarn and the interface effect with the sheath. Two more springs are applied to describe the performance of the two interlaced sheath yarns. (**b**) Illustration of Model-II, where except for a non-linear spring that is used to describe the behavior of the inner sheath yarn, other elements are kept identical to Model-I. (**c**, **d**) Comparison of the experimental and predicted results by the two theoretical models

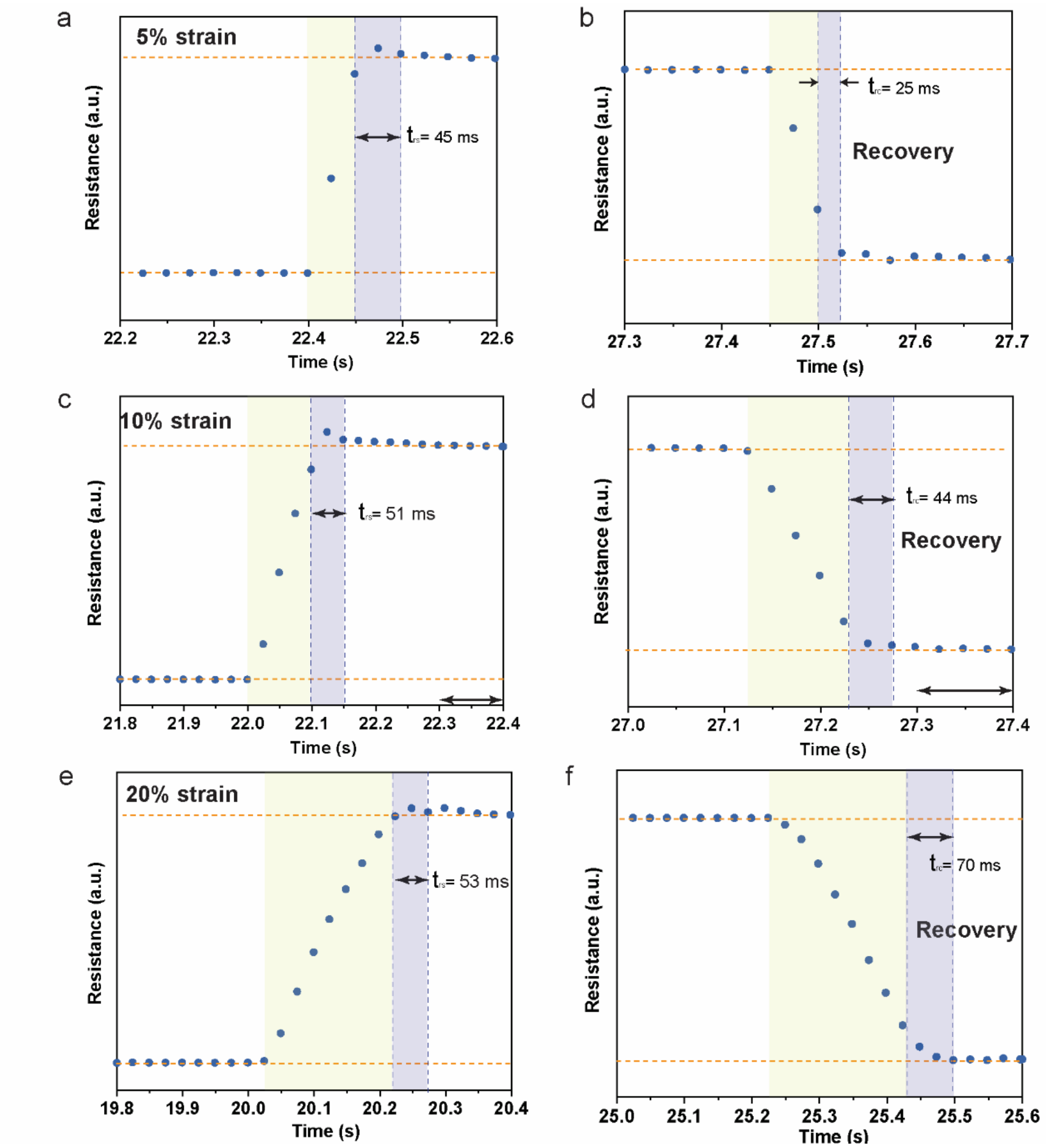

Fig. S9 Sensor response and recovery times when being stretched into different strains

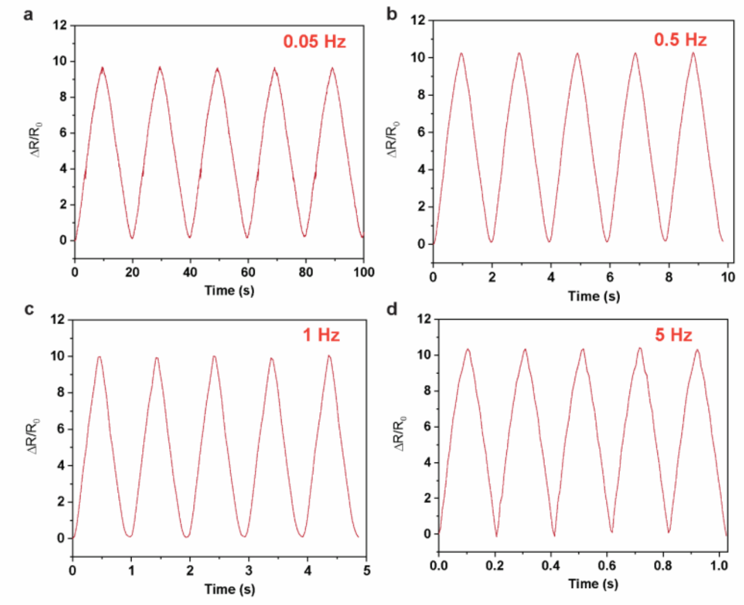

Fig. S10 Sensor response under different mechanical stretching frequencies (0.05 Hz - 5 Hz)

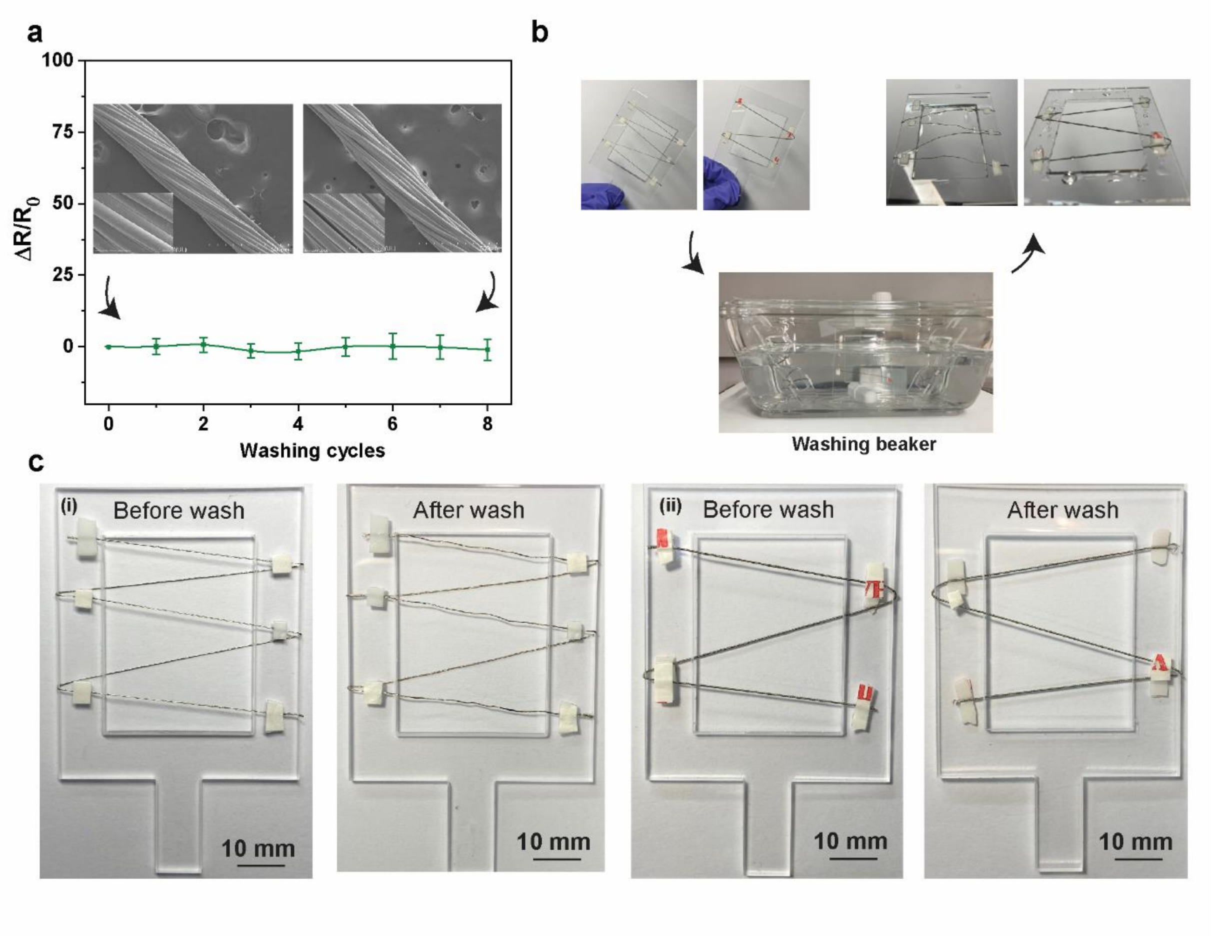

Fig. S11 Washability test of the AIYS and sheath conductive yarns. (**a**) Resistance changing rate of the sheath yarns under repeated washing test. (**b**) Pictures show the yarns before and after washing in the beaker. (**c**) Sample images of the sheath yarn and AIYS before and after wash

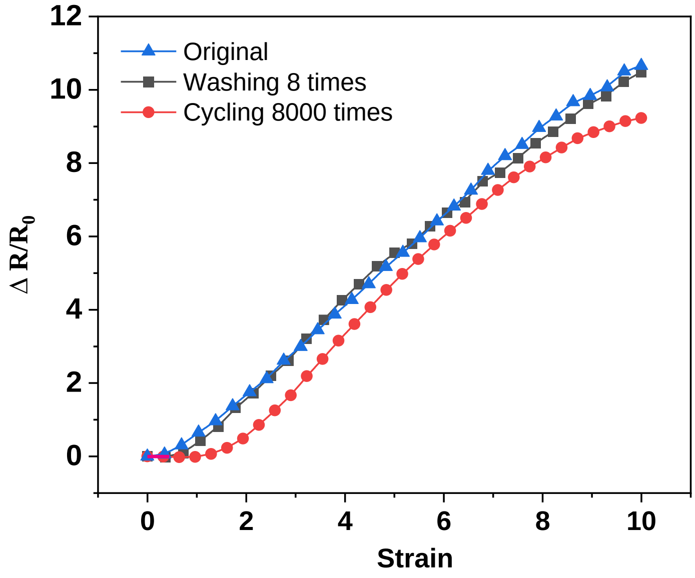

Fig. S12 Performance comparison of the AISY in the state of origin, washing 8 times, recycling for 8000 times

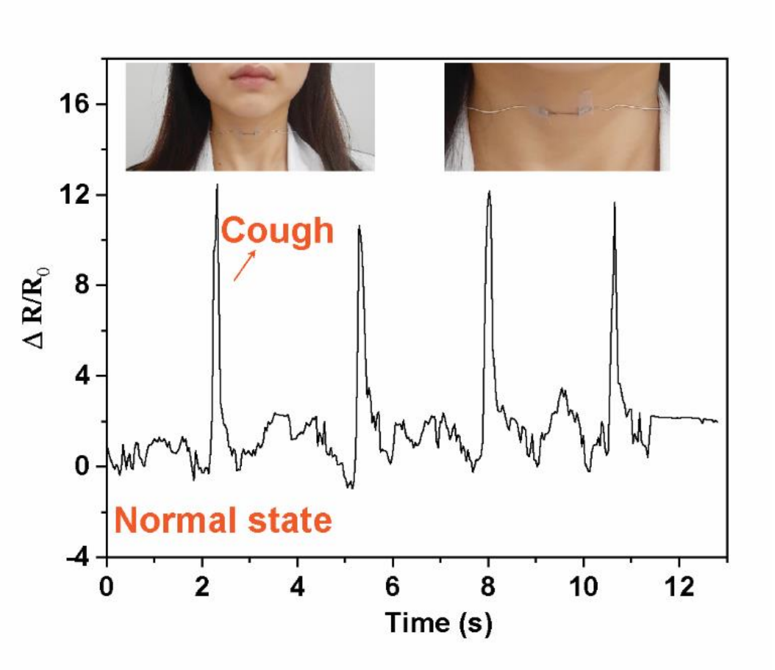

Fig. S13 Signals for detecting the human coughs when the volunteer wear a AIYS in the throat position on the neck

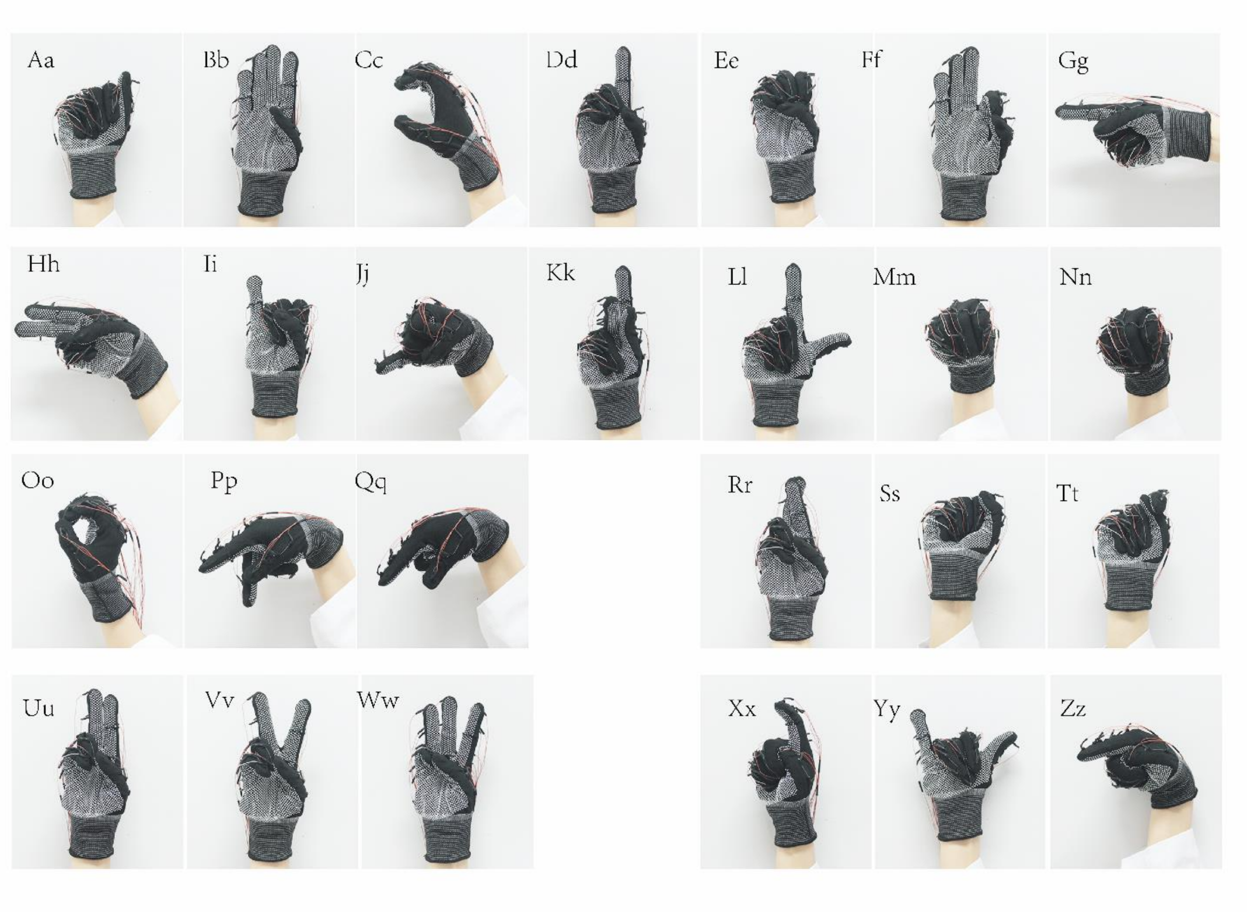

Fig. S14 Pictures showing the sign-language gestures of 26 letters of the English alphabet made by the volunteer according to ASL when wearing the smart glove with 16 AIYSs

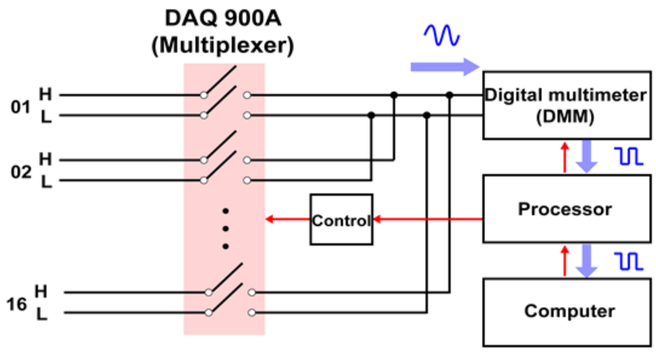

Fig. S15 Circuit diagram showing the multichannel data acquisition in the sign-language translation glove. The data flow is shown by the blue arrows. Analog signals are processed into digital signals using a digital multimeter. The controlling process is shown by the red arrows

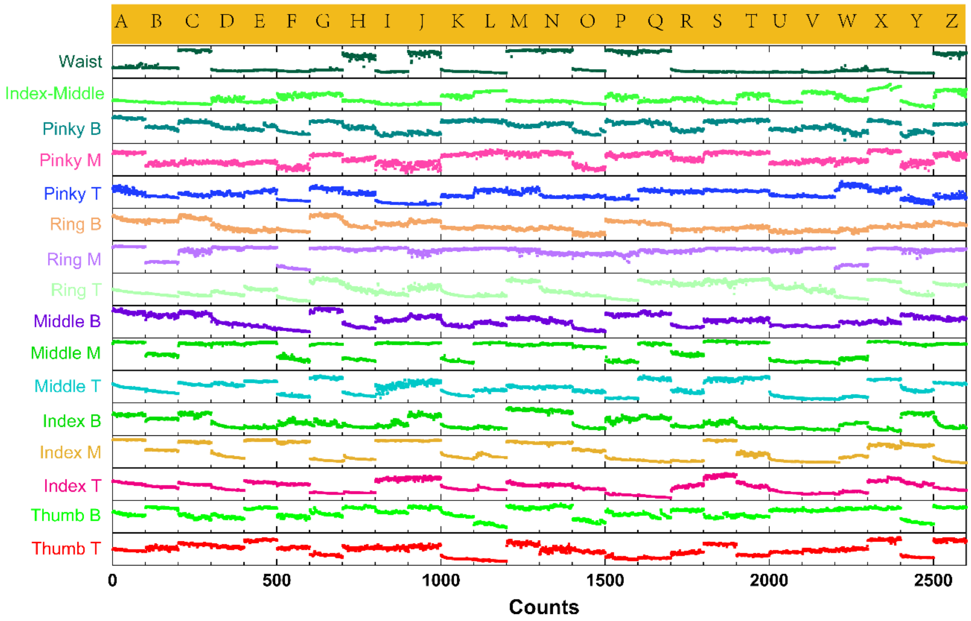

Fig. S16 Normalized raw sensor data for the sign language of the 26 letters of the English alphabet

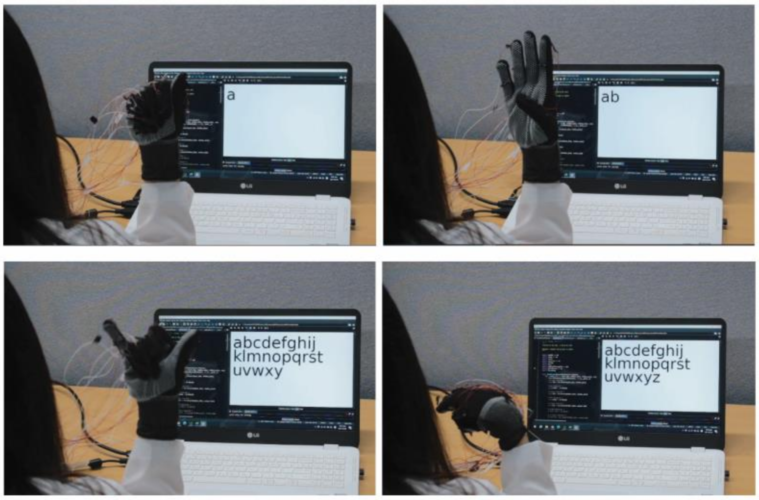

Fig. S17 Pictures showing alphabet sign language translation based on the smart glove and deep learning model

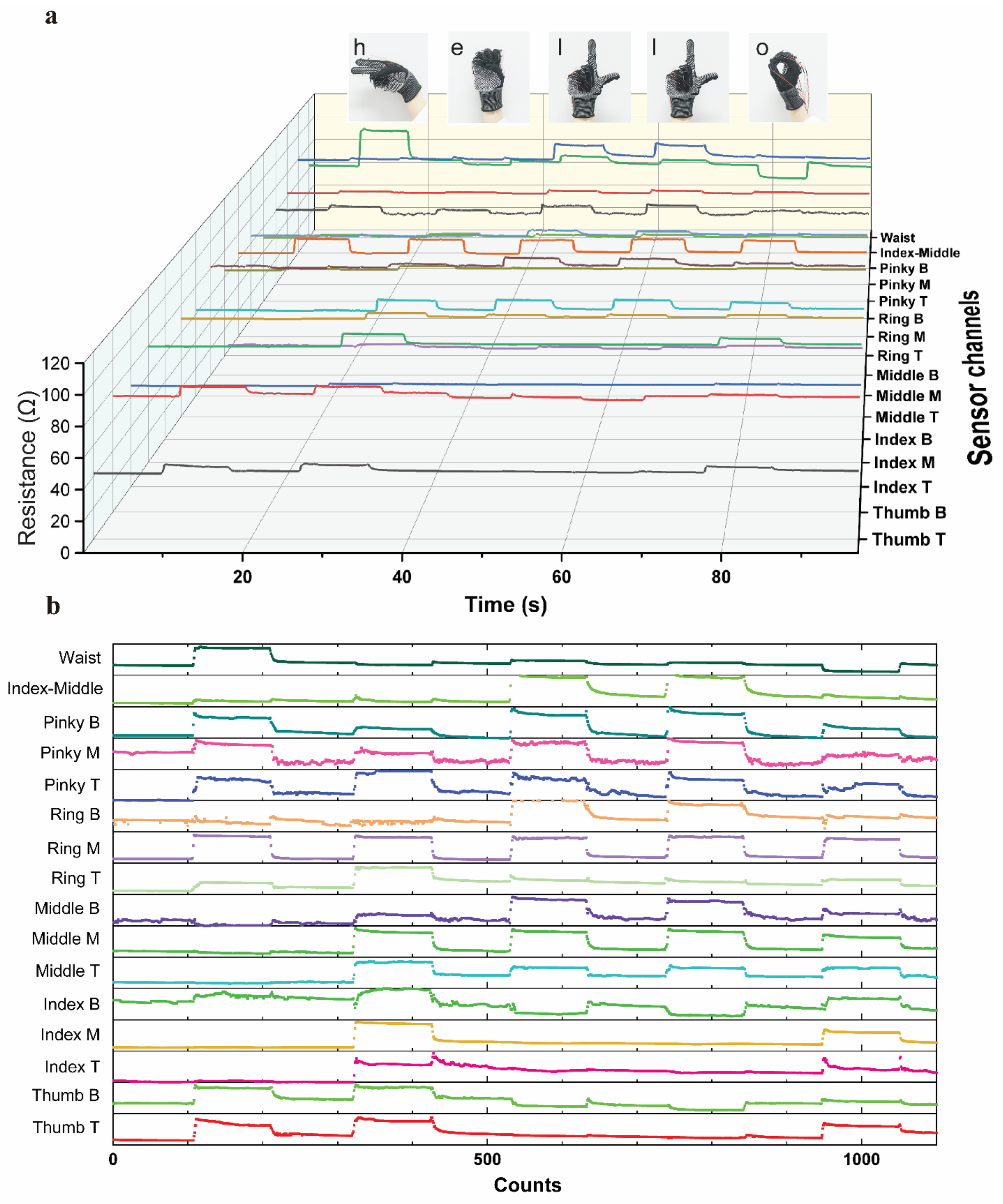

Fig. S18 Real-time resistance data (**a**) and normalized data (**b**) when the smart glove makes sign language gestures for "hello." Insets are the pictures of the letter gestures that correspond to the electrical signals

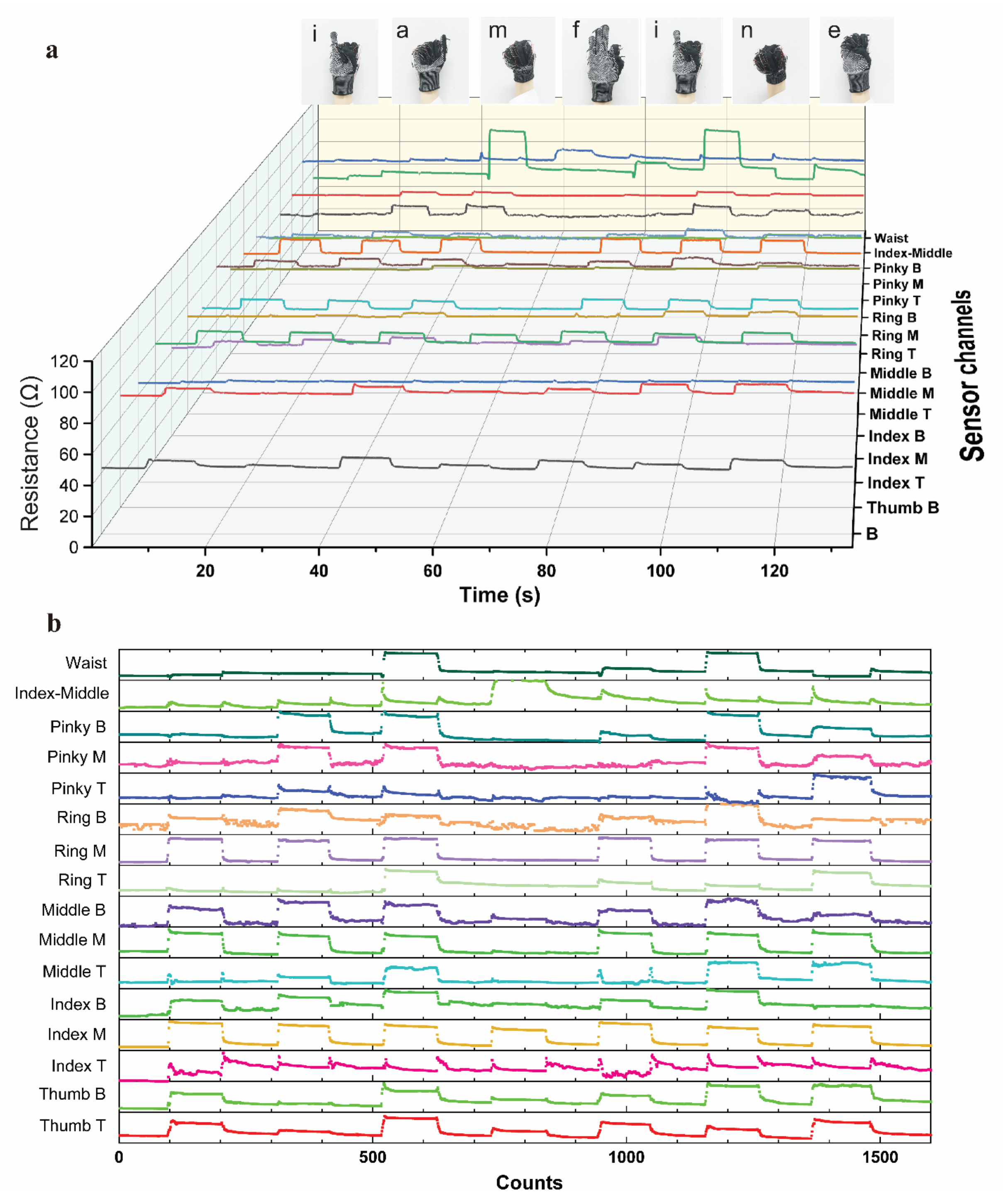

Fig. S19 Real-time resistance data (**a**) and normalized data (**b**) when the smart glove makes sign language gestures for "I am fine." Insets are the pictures of the letter gestures that correspond to the electrical signals

Supplementary References

[S1] Z.Q. Du, M. Zhou, H.L. Liu, L.G. He, Study on negative Poisson's ratio of auxetic yarn under tension: part 1-theoretical analysis. Text. Res. J. **85**, 487-498 (2015). http://doi.org/10.1177/0040517514549985

[S2] Z.Q. Du, M. Zhou, L.G. He, H.L. Liu, Study on negative Poisson's ratio of auxetic yarn under tension: part 2-experimental verification. Text. Res. J. **85**, 768-774 (2015). http://doi.org/10.1177/0040517514549987

[S3] M.H. Ussman, A.M. Manich, J. Gacen, J. Maillo, Viscoelastic behaviour and microstructural modifications in acrylic fibres and yarns as a function of textile manufacturing processing conditions. J. Text. I. **90**, 526-540 (1999). http://doi.org/10.1080/00405000.1999.10750051

[S4] A.M. Manich, M.H. Ussman, A. Barella, Viscoelastic behavior of polypropylene fibers. Text. Res. J. **69**, 325-330 (1999). http://doi.org/10.1177/004051759906900503

[S5] Y. Wang, W.D. Yu, F.M. Wang, Structural evolution and predictive modeling for nonlinear tensile behavior of tri-component elastic-conductive composite yarn during stretch. Text. Res. J. **89**, 487-497 (2019). http://doi.org/10.1177/0040517517748513
